# Is cooperation favored by horizontal gene transfer?

**DOI:** 10.1093/evlett/qrad003

**Published:** 2023-04-24

**Authors:** Thomas W Scott, Stuart A West, Anna E Dewar, Geoff Wild

**Affiliations:** Department of Biology, University of Oxford, Oxford, United Kingdom; Department of Biology, University of Oxford, Oxford, United Kingdom; Department of Biology, University of Oxford, Oxford, United Kingdom; Department of Mathematics, University of Western Ontario, London, Ontario, Canada

**Keywords:** cooperation, altruism, plasmid, horizontal gene transfer, population genetics, evolutionary theory

## Abstract

It has been hypothesized that horizontal gene transfer on plasmids can facilitate the evolution of cooperation, by allowing genes to jump between bacteria, and hence increase genetic relatedness at the cooperative loci. However, we show theoretically that horizontal gene transfer only appreciably increases relatedness when plasmids are rare, where there are many plasmid-free cells available to infect (many opportunities for horizontal gene transfer). In contrast, when plasmids are common, there are few opportunities for horizontal gene transfer, meaning relatedness is not appreciably increased, and so cooperation is not favored. Plasmids, therefore, evolve to be rare and cooperative, or common and noncooperative, meaning plasmid frequency and cooperativeness are never simultaneously high. The overall level of plasmid-mediated cooperation, given by the product of plasmid frequency and cooperativeness, is therefore consistently negligible or low.

## Introduction

Hamilton’s Rule provides a framework for understanding when cooperation evolves (Hamilton, 1963, 1964a, 1964b; West et al., 2021). It states that cooperation will evolve if the benefit of cooperation accrued by a recipient, *b*, is greater than the cost incurred by an actor, *c*, where the benefit *b* is weighted by the relatedness, *r*, between the actor and recipient, leading to *rb* > *c*. Individuals are said to be related (*r* > 0) if they share genes (genetic similarity). The most straightforward way to achieve positive relatedness (*r* > 0) is through common descent (kinship) (Grafen, 1985, 1990; Hamilton, 1963, 1964a, 1964b). That is, individuals who share a common ancestry, for example, by being siblings or cousins, may share genes that have been inherited from a common ancestor, like their mother or grandmother. The idea that common descent (kinship) facilitates the evolution of cooperation is known as *kin selection* (Hamilton, 1963, 1964a, 1964b; Maynard Smith, 1964; West et al., 2021).

It has been hypothesized that horizontal gene transfer of plasmids, or other mobile genetic elements, can increase relatedness at cooperative loci, providing another way for cooperation to evolve (Bakkeren et al., 2022; Dimitriu et al., 2014; Ghoul et al., 2017; Lee et al., 2022; Mc Ginty & Rankin, 2012; Mc Ginty et al., 2011, 2013; Nogueira et al., 2009, 2012; Rankin et al., 2011b, 2011a; Smith, 2001; West et al., 2006). The idea is that plasmid transfer increases relatedness by allowing genes to jump between bacteria that are social partners. This transfer of cooperative genes may mean that individuals are more genetically similar (related) at plasmid loci compared to chromosome (immobile) loci. Consequently, cooperation may sometimes be favored at plasmid loci even when it is disfavored at chromosomal loci, facilitating the evolution of cooperation. Horizontal gene transfer could be particularly important for facilitating cooperation in bacterial populations where there is little common ancestry between interacting cells (mixed populations).

However, there are three theoretical issues with the hypothesis that plasmid transfer facilitates cooperation. Firstly, plasmid replication is initiated at a particular DNA sequence called an origin of replication, and plasmids with an identical origin of replication compete with each other for the cell’s replication machinery. Consequently, plasmids that have a common origin of replication cannot stably coexist in a bacterial cell, even if they have different genes otherwise (plasmid incompatibility) (Novick, 1987). One implication of this is that bacterial cells cannot receive new plasmids by horizontal gene transfer if they already have a plasmid with the same origin of replication (incompatible plasmid). This affects relatedness at plasmid loci, because cooperative genes cannot be horizontally transferred between social partners if the recipient of cooperation already has its own (incompatible) plasmid. Consequently, relatedness at plasmid loci will depend on the population frequency of the plasmid (Dewar et al., 2021; Mc Ginty et al., 2013). If a plasmid is rare, meaning most individuals lack the plasmid, plasmids may be transferred among social partners, meaning social groups are likely to be genetically homogenous at plasmid loci (highly related), as hypothesized. However, if plasmids are common, meaning few individuals lack the plasmid, there may be very little plasmid transfer among social partners, because most individuals will already have the plasmid, meaning they will often be unable to acquire their social partner’s plasmid (plasmid incompatibility). The spread of plasmids through a population may therefore lead to a reduction in plasmid relatedness, meaning cooperation is not maintained in the long run. Previous theory has only examined plasmid relatedness in special cases where the plasmid is either very rare or very common or very slowly transferred (Dewar et al., 2021; Mc Ginty et al., 2013; Nogueira et al., 2009).

Secondly, a further implication of plasmid incompatibility is that cooperator plasmids cannot stably coexist in cells with plasmids that are otherwise identical but do not have the cooperation gene (Bakkeren et al., 2022; Novick, 1987). One consequence of this is that, if a plasmid that is at fixation in the population (every cell has it) acquires a mutation that causes it to cooperate, the resulting cooperator plasmid will not be able to spread, because there are no plasmid-free cells available to infect. A further consequence is that, even if a cooperator plasmid is able to invade a population, it may be outcompeted by incompatible defector plasmids that are generated via a loss-of-function mutation to the plasmid cooperation gene (Bakkeren et al., 2022; West et al., 2006). This means that plasmids might not be evolutionarily stable in conditions where they could initially invade. Previous theory has mainly focused on the invasion of plasmid-mediated cooperation, or on the special case where defector plasmids cannot arise (Mc Ginty et al., 2013; Nogueira et al., 2009; Smith, 2001). Theory is required that examines the stable maintenance of cooperator plasmids when they are in competition with defector (cheat) plasmids, as well as their initial invasion (Dewar et al., 2021).

Thirdly, even if horizontal gene transfer facilitates cooperation, leading to cooperative plasmids, this may not actually have much effect on the amount of cooperation in the bacterial population. For instance, if a cooperative plasmid resides at a low population frequency, then only a relatively low number of cells in the population will be induced to be cooperative, with the vast majority of cells remaining uncooperative. For plasmid transfer to facilitate cooperation in any appreciable sense, it is not sufficient to demonstrate that plasmids evolve to be cooperative. We would also need to demonstrate that the cooperative plasmids evolve to an appreciable frequency in the population. Previous theoretical work has examined what frequency plasmids evolve to, but has not explicitly tied this to an analysis of how cooperative the bacterial population is therefore likely to be as a whole (Bergstrom et al., 2000; Dewar et al., 2021; Smith, 2001).

We address these issues theoretically by examining the coevolution of plasmid- and chromosome-mediated cooperation. We calculate: (1) an explicit plasmid relatedness function, which allows us to see when plasmids evolve to be cooperative, both initially and at equilibrium; (2) the frequency that plasmids evolve to at equilibrium. By jointly considering the cooperativeness and frequency of plasmids, we can examine what effect plasmids have on the overall level of cooperation in bacterial populations. Finally, we examine the extent to which evolutionary theory can explain the empirical data for cooperation on plasmids.

## Methods

We adopted a model developed by Dewar et al. (2021) (same lifecycle assumptions), which in turn generalized a simpler model developed by McGinty et al. (2013); Dewar et al.’s (2021) model examines when cooperation evolves when it may be encoded by a plasmid or the bacterial chromosome. We extended Dewar et al.’s (2021) analysis by explicitly deriving plasmid relatedness and examining plasmid frequency.

We assumed an infinite population of haploid individuals (bacterial cells). Individuals may carry a cooperative gene that codes for public goods production, on a plasmid or the chromosome or both (redundancy). We also allowed for the possibility of: noncooperative plasmids and chromosomes; plasmid-free cells; and a cost of plasmid carriage (*C*_*C*_). In each generation, the population is divided into patches, each founded by *N*-independent cells. Cells reproduce clonally until there is a large number of cells per patch. Each cell then finds a partner, and if a plasmid-free individual has a plasmid-bearing partner, with probability β, the plasmid-free individual acquires a copy of its partner’s plasmid (horizontal gene transfer). Individuals with a gene for cooperation then produce a public good, at a cost *C*_*G*_, which generates a benefit *B* that is shared between all members of the patch. Individuals then survive according to their fitness. Plasmid-bearing individuals lose their plasmid with probability *s*. Finally, individuals disperse to find new patches. We represent this lifecycle mathematically in Supplementary Appendix A, using population genetic difference equations (recursions). We iterated these recursions over many generations to find out when plasmids facilitate: the invasion of cooperation; the maintenance of cooperation at evolutionary equilibrium.

Our general approach was as follows. First, we examined how plasmid relatedness changes through the course of plasmid evolution, which allowed us to examine when plasmids evolve to be cooperative. Plasmid relatedness was also examined by Mc Ginty et al. (2013) and Dewar et al. (2021) in a simpler model where plasmids cannot evolve to be cooperative. However, we show in Supplementary Appendix B that these previous relatedness expressions are only accurate for the special case where the plasmid is either very rare or common, or where the plasmid is very slowly transferred (Dewar et al., 2021; Mc Ginty et al., 2013). In Supplementary Appendix B, we extended these analyses to obtain a more general plasmid-relatedness expression, *R*_*plas*_. Second, we examined what frequency plasmids evolve to. Third, we combined the effects of plasmid relatedness and plasmid frequency to examine what overall effect plasmids had on the evolution of cooperation.

## Results

### When do plasmids evolve to be cooperative?

**Figure 1.**
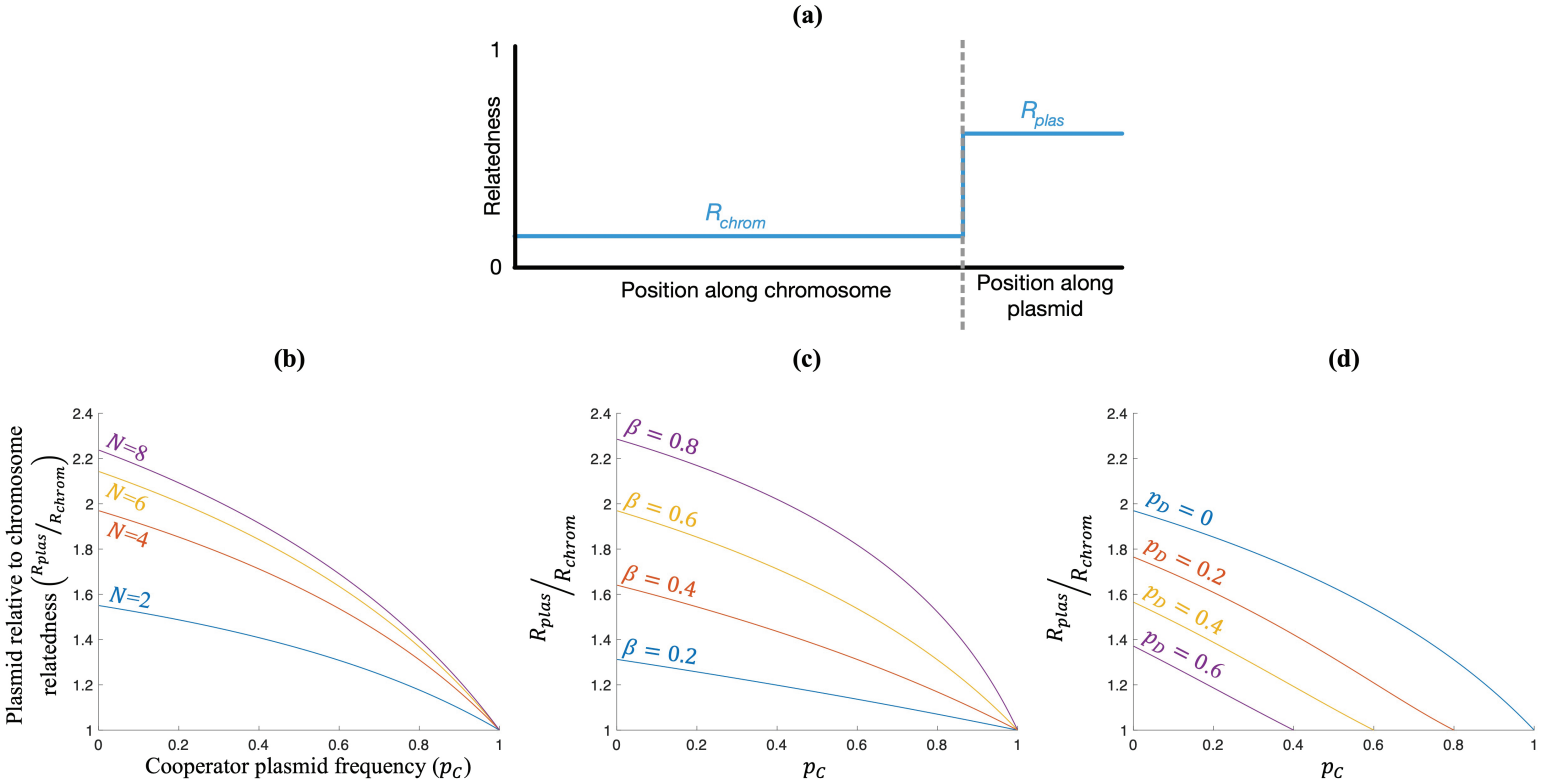
**Plasmid relative to chromosome relatedness**. (A) Horizontal gene transfer may allow relatedness at plasmid loci (*R*_*plas*_) to increase above relatedness at chromosomal loci (*R*_*chrom*_). (B–D) We plot plasmid relatedness relative to chromosome relatedness $(R_{plas}/R_{chrom}),$ where *R*_*chrom*_ = 1/*N* as a function of the population frequency of the cooperator plasmid (*p*_*C*_). We vary the: (B) number of founder cells per group (*N*); (C) plasmid transfer probability (*β*); and (D) defector plasmid frequency (*p*_*D*_). When plasmids are at fixation (*p* + *p*_*D*_ = 1), plasmid relatedness equals chromosome relatedness $\left(R_{plas}/R_{chrom}=1\right)$. (B–D) We assumed *N* = 6, *β* = 0.6, and *p*_*D*_ = 0 except where specified.

We derive in Supplementary Appendix B a fully explicit expression for plasmid relatedness (*R*_*plas*_). We found that plasmid relatedness decreases monotonically with plasmid frequency (Figure 1; Supplementary Figure 1). When plasmids are exceedingly rare, which is the case when the plasmid first invades a population, transfer on a plasmid substantially increases relatedness. Relatedness is substantially increased because there are many plasmid-free cells available, meaning plasmids have many opportunities to be transferred, generating genetic similarity (*R*_*plas*_ > *R*_*chrom*_). This means that horizontal gene transfer can increase the likelihood of Hamilton’s Rule being satisfied (*R*_*plas*_*B* > *C*_*G*_), helping cooperation invade (Dimitriu et al., 2016; Mc Ginty & Rankin, 2012; Mc Ginty et al., 2011, 2013; Nogueira et al., 2009; Rankin et al., 2011a). Plasmid relatedness is higher if they are transferred with greater probability (higher *β*), leading to greater genetic similarity between social interactants (Figure 1).

As plasmids become more common, when they spread through the population, transfer on a plasmid causes a smaller increase in relatedness (Dewar et al., 2021) (Figure 1). Relatedness is lower because there are fewer plasmid-free cells available, meaning plasmids have fewer opportunities to be transferred, reducing the extent to which transfer increases relatedness. This means that, as a plasmid spreads through a population, the likelihood of Hamilton’s Rule being satisfied (*R*_*plas*_*B* > *C*_*G*_) is reduced. If plasmid relatedness (*R*_*plas*_) falls enough that Hamilton’s Rule is no longer satisfied, plasmids evolve to no longer cooperate at equilibrium (Dewar et al., 2021).

**Figure 2.**
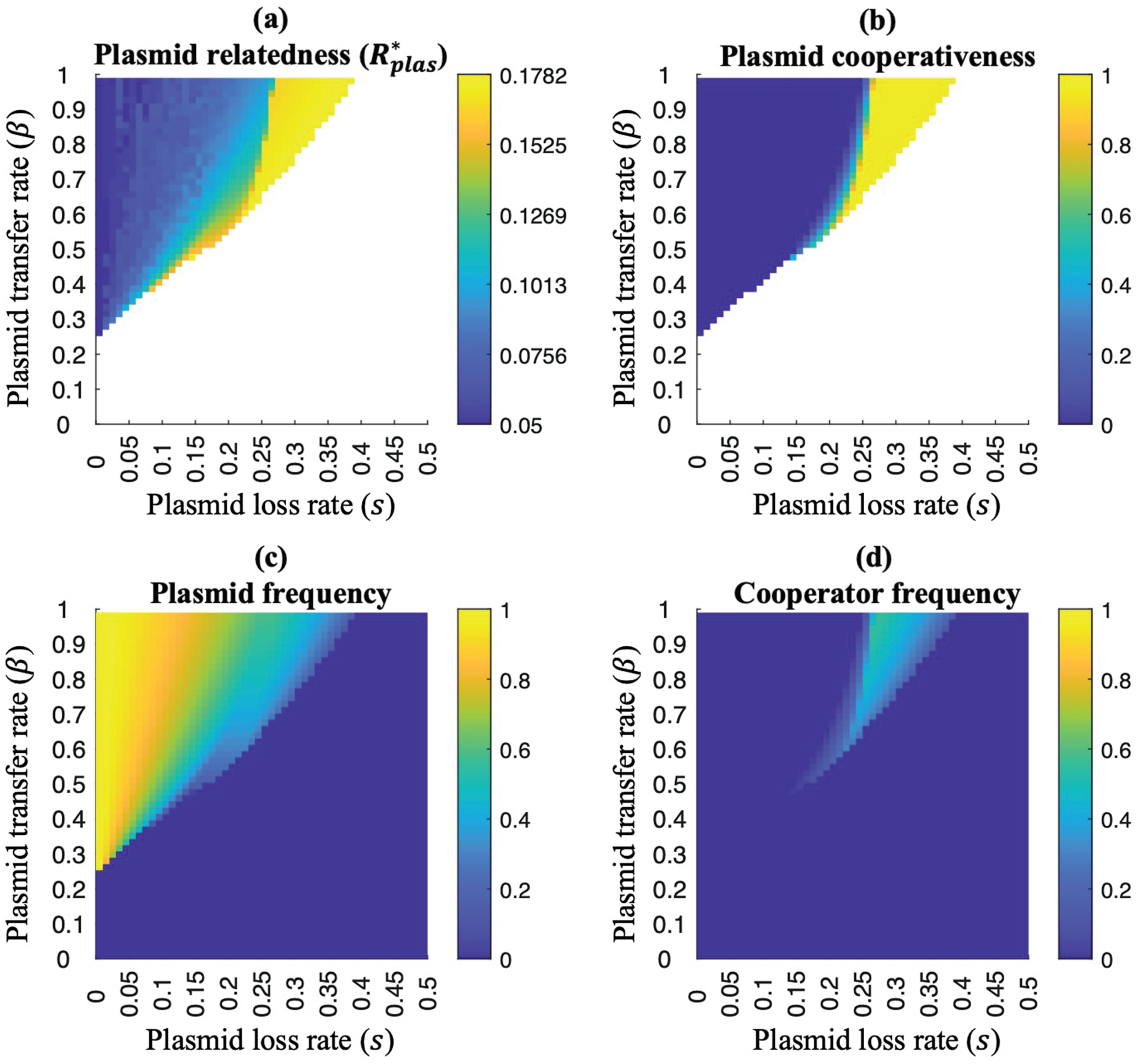
**Plasmid-mediated cooperation**. Example results for a region of parameter space where cooperation is not favored at the chromosome (individual) level (*R*_*chrom*_*B* < *C*_*G*_), meaning cooperation only evolves if it is encoded by a plasmid. We record the equilibrium: (A) plasmid relatedness (Equation 29); (B) plasmid cooperativeness (proportion of plasmids that are cooperative; we find that this is 1 for $R_{plas}^{*}B>C_{G}$ and 0 for $R_{plas}^{*}B<C_{G}$); (C) plasmid frequency (including both cooperator and defector plasmids); (D) cooperator frequency (given by the product of plasmid cooperativeness and frequency). (A–D) We assumed: *N* = 20, *C*_*C*_ = 0.2, *C*_*G*_ = 0.1, and *B* = 1.435. The white areas in A and B represent areas of parameter space that are undefined because plasmids are absent. The lowest recorded plasmid relatedness in A is equal to chromosomal relatedness (0.05 = 1/*N*).

In the absence of plasmid loss (*s* = 0), plasmids that invade go to fixation at equilibrium, and cooperation is only favored when *R*_*chrom*_*B* > *C*_*G*_, where *R*_*chrom*_ is the genetic relatedness at the chromosomal (individual) level (*R*_*chrom*_ = 1/*N*) (Figure 2A). Chromosomal relatedness (*R*_*chrom*_) is generated as a consequence of population structure, where fewer founder cells on each patch (*N*) corresponds to a more structured (viscous) population. Cooperation is therefore only favored on the plasmid when it provides a kin-selected benefit at the level of the chromosome (individual) (Dewar et al., 2021; Mc Ginty et al., 2013). The reason for this result is that, in the absence of plasmid loss (*s* = 0), plasmids continue to increase in frequency after invasion, ultimately reaching fixation in the population. At this equilibrium, there are no plasmid-free individuals left to infect, which means that the overall level of horizontal gene transfer in the population goes to zero. Consequently, competition between plasmids with and without a cooperative gene (cooperators and cheats) becomes analogous to the scenario in which the gene for cooperation is on the chromosome (Dewar et al., 2021; Mc Ginty et al., 2013) (Figure 2B).

When plasmids can be lost (*s* > 0), this can favor long-term cooperation on plasmids, but only if plasmids are transferred rapidly (high β) and are lost quickly (high *s*) (Figure 2A). Plasmid loss means that plasmids do not reach fixation in the population and so plasmid transfer can still occur in the evolutionary long term. This increases relatedness at the cooperative plasmid locus relative to the chromosomal locus, which may favor cooperation on the plasmid, when it would not otherwise be favored on the chromosome (technically, this will be the case when:$R_{plas}^{*}>(C_{G}/B)>R_{chrom}^{*}=(1/N)$) (Figure 2B). An increased plasmid loss rate (*s*) increases the proportion of plasmid-free cells at equilibrium, and an increased plasmid transfer probability (β) increases the likelihood that a plasmid is transferred between social partners, both of which lead to a higher equilibrium plasmid relatedness ($R_{plas}^{*}$) and an increased chance that plasmids evolve to be cooperative (*R*_*plas*_*B* > *C*_*G*_).

One way of thinking about our results is that the relatedness for a gene on a plasmid (*R*_*plas*_) is equal to the relatedness for a gene on a chromosome (*R*_*chrom*_) plus an extra amount due to horizontal gene transfer (because plasmid relatedness builds through vertical and horizontal transmission). When plasmids are rare, there is substantial horizontal gene transfer and so relatedness at a cooperative locus is substantially increased when the locus itself is found on a plasmid. As plasmids become more common, the rate of horizontal gene transfer is reduced, and so being on a plasmid causes less of an increase in relatedness. In the extreme, when a plasmid is at fixation, there is no horizontal gene transfer and so relatedness on a plasmid is equal to the relatedness for a gene on a chromosome (*R*_*plas*_*= R*_*chrom*_). The loss of plasmids (*s* > 0) increases relatedness because it frees up bacterial cells for horizontal gene transfer.

Overall, horizontal gene transfer can help cooperation initially invade but will then often have less influence on whether cooperation is maintained in the long term. We found that this is because plasmid relatedness decreases monotonically with plasmid frequency, meaning plasmid relatedness is high at the time of invasion but is lower at equilibrium (Figure 1; Supplementary Figure 1). Our results are consistent with previous theoretical analyses that examined plasmid relatedness in special cases (Dewar et al., 2021; Mc Ginty et al., 2013).

### What frequency do plasmids evolve to?

We show in Supplementary Appendix C that plasmids can only invade if they spread faster than they are lost. Plasmids spread via horizontal gene transfer (*β*), and may be lost directly from cells (*s*). Furthermore, plasmids may spread, or be lost, through their effects on host fitness. A greater plasmid carriage cost (*C*_*C*_) makes plasmids less likely to invade. A greater kin-selected benefit to cooperation (higher *B*, lower *N*, and *C*_*G*_) makes cooperator plasmids more likely to invade, and defector plasmids less likely to invade, in populations of noncooperative bacterial hosts. Overall, if *β* is sufficiently high relative to *s* and *C*_*C*_, plasmids of at least one type (cooperator or noncooperative) will be able to invade.

**Figure 3.**
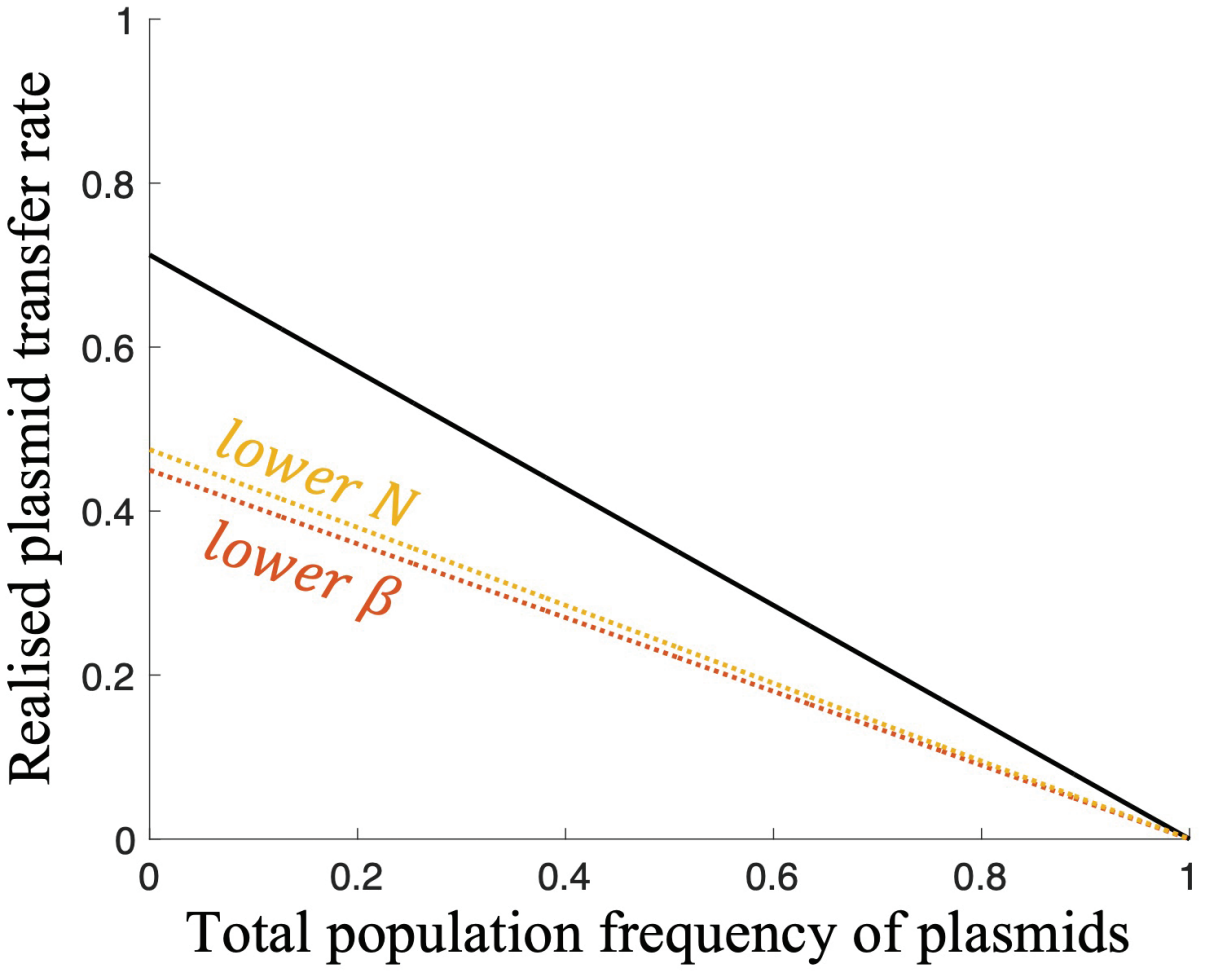
**Realized plasmid transfer rate**. We plot the realized plasmid transfer rate for a given plasmid (of any type), for different: numbers of founder cells per group (*N*), plasmid transfer probabilities (*β*). The solid black line represents the case where *β* = 0.95 and *N* = 4. The colored dashed lines represent scenarios where there is a lower number of founder cells (*N* = 2; yellow line) and a lower plasmid transfer probability (*β* = 0.6; red line).

After invasion, plasmids spread until they reach a given (equilibrium) frequency. The total equilibrium frequency of plasmids (cooperators + defectors) is higher when plasmids are transferred more quickly (*β*) relative to the rate at which they are lost (*s*), and when plasmids are less detrimental to host fitness (Figure 2C and Supplementary Figure 2). In the special case where plasmids are not directly lost (*s* = 0), plasmids that can invade will spread until all individuals in the population have a plasmid of some type (plasmid fixation) (Smith, 2001) (Figure 2C). To understand how the total equilibrium plasmid frequency is influenced by *β*, *s*, and effects on host fitness (*B*, *N*, *C*_*G*_, and *C*_*C*_), it is useful to define the *realized* plasmid transfer rate as the product of: the probability that, when a plasmid-free cell is paired with a plasmid-bearing cell, the plasmid is transferred (given by *β*); the availability (local frequency) of plasmid-free cells (given by [(N−1)/N]∗(1−total population frequency of plasmids)) (Figure 3). The realized plasmid transfer rate, just like the plasmid transfer probability parameter *β*, is the same for both cooperator and defector plasmids (same origin of replication).

When the total plasmid frequency is low, which is the case when plasmids first invade a population, the realized plasmid transfer rate is high relative to the rate of plasmid loss (Figure 3). The realized plasmid transfer rate is high because there are many plasmid-free cells available, meaning plasmids (of either type) have many opportunities to be transferred. By contrast, when the total plasmid frequency is high, which is the case when plasmids have had a chance to spread through the population, the realized plasmid transfer rate is lower (Figure 3). The realized plasmid transfer rate is lower because there are fewer plasmid-free cells available, meaning plasmids (of either type) have fewer opportunities to be transferred. Furthermore, at the population level, the rate at which plasmids are gained or lost through host fitness (mediated by *B*, *N*, *C*_*G*_, and *C*_*C*_) decreases for low and high total plasmid frequencies, where there is lower variance among members of the population for plasmid carriage, and therefore a lower efficacy of selection. Note that this is the same for any gene under selection (change in allele frequency due to selection is proportional to variance in allele frequency, meaning it is lower for low and high allele frequencies) (Fisher, 1930; Price, 1970, 1972). Therefore, as plasmids (of either type) spread through the population, the overall rate at which new plasmids (of either type) are added to the population, whether by horizontal transfer (*β*) or by improving host fitness, will inevitably start to fall.

However, the rate at which plasmids are directly lost (*s*) does not change as plasmids (of either type) spread through the population, because this process is not modulated by the genotypic constitution of the population (such as the local frequency of plasmid-free cells). This means that there is a permanent, constant evolutionary force removing plasmids from the population (Bergstrom et al., 2000). Therefore, as plasmids (of either type) spread through a population, the overall rate at which new plasmids (of either type) are added to the population will eventually decrease relative to the rate at which they are lost. Eventually, the rate at which new plasmids (of either type) are added to the population will fall to equal the rate at which they are lost. At this point, plasmids (of either type) are lost from the population at exactly the same rate that new plasmids are added, meaning the total plasmid frequency does not change any further (dynamic equilibrium). The equilibrium total plasmid frequency is therefore mediated by the rate of plasmid transfer (*β*) and loss (*s*), as well as by the effect of plasmids on host fitness (*B*, *N*, *C*_*G*_, and *C*_*C*_). The equilibrium balance between plasmid loss (*s*) and gain (through effects on host fitness as well as horizontal transmission) is analogous to the equilibrium balance between gene loss (through mutation) and gain (by selection) that characterizes all mutable genes under positive selection (mutation-selection balance) (Bergstrom et al., 2000; Fisher, 1930).

### What effect do plasmids have on the overall level of bacterial cooperation?

The extent to which transfer on a plasmid favors higher levels of cooperation depends upon two things:

(1) The extent to which equilibrium plasmid relatedness ($R_{plas}^{*}$) is greater than chromosome relatedness $\left(R_{chrom}^{*}\right)$. In the first section of the results, we found that equilibrium plasmid relatedness ($R_{plas}^{*}$) is higher when there is appreciable horizontal gene transfer in the evolutionary long run, which is the case when there are high rates of plasmid transfer (*β*) and plasmid loss (*s*).
(2) The equilibrium frequency of plasmids in the population. In the second section of the results, we found that the total equilibrium plasmid frequency is higher when there are high rates of plasmid transfer (*β*) and low rates of plasmid loss (*s*).

We examined the overall effect of plasmids on bacterial cooperation by combining these two results. We found the overall level of cooperation in the population (Figure 2D) by multiplying equilibrium plasmid cooperativeness (proportion of plasmids that are cooperative; Figure 2B) by the equilibrium total plasmid frequency (Figure 2C). When cooperation is favored at the chromosome (individual) level (*R*_*chrom*_*B* > *C*_*G*_), cooperation evolves on the chromosome, meaning plasmids trivially have no effect on the overall level of cooperation (redundancy) (Dewar et al., 2021).

**Figure 4.**
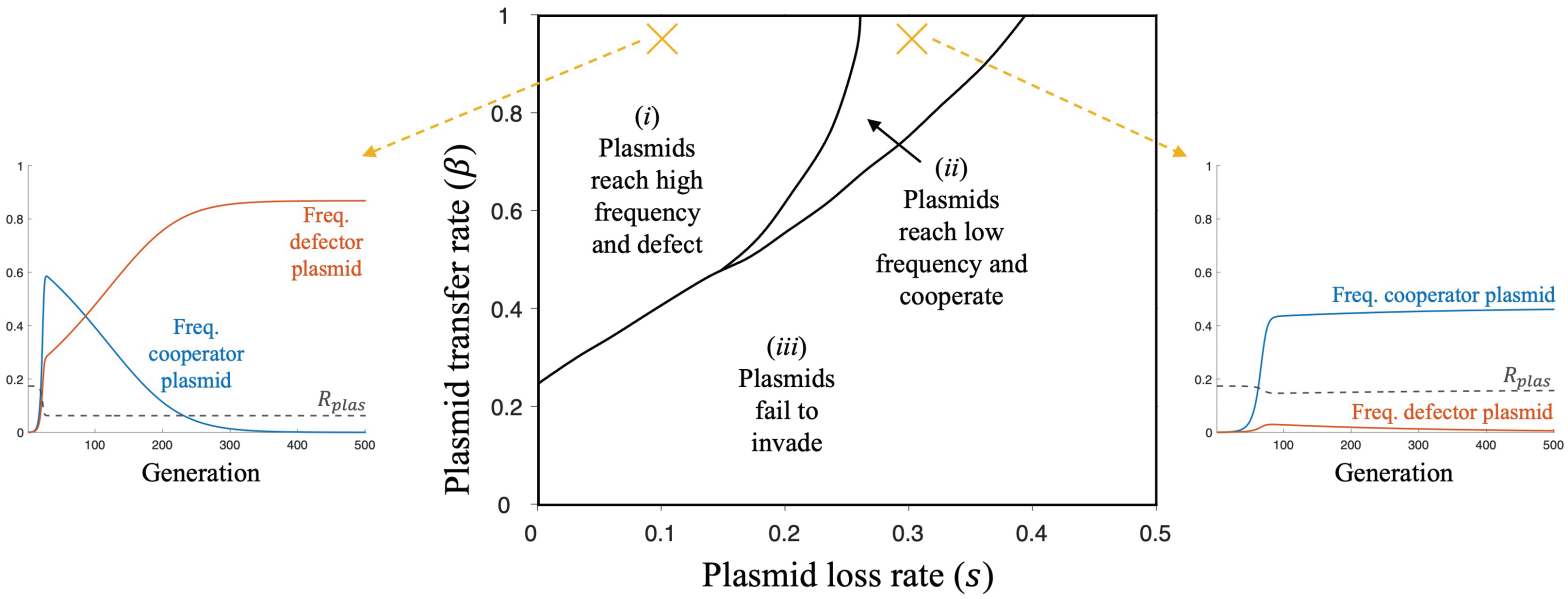
**Plasmids do not appreciably increase the amount of cooperation at equilibrium**. Example results for a region of parameter space where cooperation is not favored at the chromosome (individual) level (*R*_*chrom*_*B* < *C*_*G*_), meaning cooperation only evolves if it is encoded by a plasmid. We assumed: *N* = 20, *C*_*C*_ = 0.2, *C*_*G*_ = 0.1, and *B* = 1.435 (same as Figure 2). “Low” and “high” plasmid frequencies in this figure are taken to be <0.58 and >0.58, respectively. Dashed yellow lines lead to example trials (*β* = 0.95, *s* = {0.1,0.3}), plotting cooperator, and defector plasmid frequencies, alongside plasmid relatedness (*R*_*plas*_), over time.

When cooperation is not favored at the chromosome (individual) level (*R*_*chrom*_*B* < *C*_*G*_), cooperation only evolves if it is encoded by a plasmid. In these cases, we obtained two results. Firstly, we found that high levels of cooperation do not arise at evolutionary equilibrium. The reason is that plasmid relatedness and plasmid frequency cannot be simultaneously large, owing to a trade-off mediated by the plasmid loss rate (*s*) (Figures 2D and 4). Specifically, a high rate of plasmid loss (*s*) is necessary to generate high equilibrium plasmid relatedness, but a low rate of plasmid loss (*s*) is necessary to generate high total equilibrium plasmid frequency—the two quantities cannot be simultaneously maximized.

Secondly, we found that while they are still relatively low, the highest levels of cooperation can be found in a window of parameter space, characterized by a high rate of plasmid transfer (*β*) alongside intermediate plasmid loss (*s*) and a low number of founders (*N*) (Figures 2D and 4). A high rate of plasmid transfer (*β*) leads to increased equilibrium plasmid relatedness ($R_{plas}^{*}$) and frequency, facilitating cooperation. An intermediate plasmid loss rate (*s*) corresponds to a sweet spot where plasmid relatedness remains elevated over chromosomal relatedness in the evolutionary long run $\left(R_{plas}^{*}>R_{chrom}^{*}=1/N\right)$, but plasmids are not pushed down to a negligible total population frequency. A low number of founders (*N*) leads to an increased “background” relatedness (population structuring/ viscosity), which increases the likelihood that all genes (not just plasmid genes) are cooperative, and does so without necessarily reducing the total equilibrium plasmid frequency (Supplementary Figure 2).

This “goldilocks” combination of parameter values, characterized by high *β*, intermediate *s* and low *N*, may facilitate the evolution of low/intermediate levels of bacterial cooperation in some natural bacterial populations. However, it cannot explain the evolution of bacterial cooperation in any general sense, where *β*, *s*, and *N* may vary outside of this restrictive area of parameter space.

## Discussion

We found that plasmids have little influence on the overall level of cooperation in bacterial populations (Figure 4). This is because plasmid relatedness and plasmid frequency are highest in different areas of parameter space, meaning both cannot be simultaneously high (Figure 2A and C). On the one hand, for plasmids to reach high frequency, they can’t be lost too quickly, for instance, by segregation at the point of cell division (Figure 2C). On the other hand, for plasmids to achieve high relatedness and become cooperative, they need to be lost quickly, so that plasmid-free cells are always available for the plasmid to jump into (Figure 2A). Consequently, as the plasmid loss rate (*s*) increases, plasmids evolve to a lower frequency and higher relatedness (cooperativeness). The overall level of bacterial cooperation is therefore relatively unaffected by plasmids because, for any given rate of plasmid loss (*s*), plasmids are either too rare or too uncooperative to have much effect (Figure 4). Plasmids have their largest, but still relatively small, effect on bacterial cooperation in a “goldilocks” region of parameter space where: the plasmid loss rate (*s*) is intermediate, the rate of plasmid transfer (*β*) is high, and the population is already relatively highly related due to population structuring/ viscosity (low *N*) (Figure 2D).

Our analysis often referred to long-term evolutionary states (equilibria), but this may not be appropriate for many plasmids. In nature, many plasmids are likely to be in constant flux, subject to environments (and associated selection pressures) that are constantly changing. Consequently, many plasmids may not converge on an equilibrium state with a stable frequency and relatedness (cooperativeness). However, even plasmids that are pulled away from evolutionary equilibria will not be able to appreciably influence bacterial cooperation. The reason for this is that plasmids that lie away from equilibria will still be constrained by the trade-off between plasmid frequency and relatedness. Specifically, to achieve high frequency, a plasmid needs to be lost slowly, and to achieve high relatedness (cooperativeness), it needs to be lost quickly enough that there are plasmid-free cells to infect. This is true whether or not the plasmid is in a stable environment (evolutionary equilibrium) or faces environmental flux (nonequilibrium) (Supplementary Appendix E).

Our discussion focused on the hypothesis that plasmids may increase bacterial cooperation by increasing relatedness (“relatedness hypothesis”) (Dewar et al., 2021; Mc Ginty & Rankin, 2012; Mc Ginty et al., 2011, 2013; Nogueira et al., 2009; Rankin et al., 2011a). An alternative hypothesis is that plasmids may increase bacterial cooperation by infectivity (Smith, 2001). The idea here is that noncooperators may be “infected” by the cooperative plasmid, changing them back into cooperators, meaning cooperation is “enforced” and persists at equilibrium (“infectivity hypothesis”) (Giraud & Shykoff, 2011; Lee et al., 2022; Smith, 2001). Our model captures the influence of both relatedness and infectivity (Mc Ginty et al., 2013). We found that the infectivity hypothesis, like the relatedness hypothesis, may allow cooperation to invade (Dewar et al., 2021; Mc Ginty et al., 2013; Rankin et al., 2011a). However, we found that infectivity does not allow cooperation to be maintained in the long term, because defector plasmids will arise by loss-of-function mutation of the cooperator plasmid, and will infect plasmid-free cells just as quickly as the cooperator plasmid, meaning cooperation is no longer enforced (Rankin et al., 2011a). A further problem with the infectivity hypothesis is that it works for any trait, allowing any trait to spread, such as being colored red, or swimming in circles. It does not explain why cooperation should be enforced by plasmids more than anything else. Neither the relatedness nor infectivity hypothesis is rescued by population structure, as can be the case with greenbeards, because selection on plasmids is negative not positive frequency-dependent (Gardner & West, 2010; Supplementary Appendix D).

One factor we omitted from our models is the potential for evolutionary conflict with the bacterial chromosome (Ghoul et al., 2017; Queller & Strassmann, 2018; West et al., 2006). We found that plasmids may facilitate cooperation by increasing the level of cooperation above what is preferred by the bacterial chromosome $[R_{plas}>(C_{G}/B)>R_{chrom}].$ However, this increase in cooperation will be opposed by the chromosome, with mutations arising on the chromosome that suppress plasmid cooperation (Ågren et al., 2019; Gardner & Úbeda, 2017; Mc Ginty & Rankin, 2012; Scott & West, 2019). By not accounting for such mutations, our results are the upper limit for, and therefore likely overestimate the extent that plasmids can increase bacterial cooperation (Mc Ginty & Rankin, 2012). While it is true that plasmids may evolve counter-adaptations to re-instate cooperation, individual plasmids (plasmid coreplicons) are generally much smaller than the chromosome, meaning they generate adaptive mutations at a lower rate (Cosmides & Tooby, 1981; Dewar et al., 2021). This means that plasmids are likely to wield less power than the chromosome in the arms race over the level of cooperation (Grafen, 2006; Queller & Strassmann, 2018; Scott & Queller, 2019; Scott & West, 2019). The “parliament of genes” principle, therefore, predicts that cooperation would evolve to be even lower than predicted by our model, in accordance with the evolutionary interest of the bacterial chromosome (Ågren, 2021; Leigh, 1971; Scott & West, 2019). Explicit theory addressing these issues would be very useful.

To conclude, our model is supported by and can explain the empirical observation that genes for cooperative behaviors are not more likely to be found on plasmids. Dewar et al. (2021) carried out a comparative study across 51 bacterial species examining genes for extracellular proteins, that were likely to act as cooperative public goods. They found that these cooperative genes were not more likely to be carried on: (1) plasmids compared to chromosomes; (2) plasmids that transfer at higher rates (higher *β*). These results are consistent with our prediction that plasmids will have little influence on the overall level of cooperation in bacterial populations (Figure 4). In addition, our model can explain experiments carried out by Bakkeren et al. (2022), which showed that that location on a conjugative plasmid can help a cooperative trait invade in *Salmonella enterica* serovar Typhimurium (S.Tm) but that this was only stable with strong population bottlenecks (high relatedness). We are not saying that horizontal gene transfer can never favor cooperation, but rather that, on average, horizontal gene transfer on plasmids has not consistently favored cooperation.

## Supplementary material

Supplementary material is available online at *Evolution Letters* This includes Supplementary Appendices A, B, C, D & E, and Supplementary Figures 1 & 2.

qrad003_suppl_Supplementary_Material

## Data Availability

The data generated during the current study are available at https://doi.org/10.5281/zenodo.7585371 (Scott et al., 2023). We include programs for: implementing our mathematical model; testing our relatedness expression (Equation 29) with simulation; generating figures.
