# Supplementary material for: Is cooperation favored by horizontal gene transfer?
Source: Evol Lett. 2023 Apr 24;7(3):113–20. doi: 10.1093/evlett/qrad003 (PMC10210433; doi:10.1093/evlett/qrad003)
Supplement: qrad003_suppl_Supplementary_Material [file qrad003_suppl_supplementary_material.pdf]

Supplementary information for 'Is cooperation favoured by horizontal gene transfer?'

This supplementary document contains:

p1) Appendix A: Mathematical model.

p16) Appendix B: Derivation of plasmids relatedness in terms of model parameters.

p30) Appendix C: Invasion criteria.

p33) Appendix D: Plasmids versus greenbeards.

p35) Appendix E: Equilibria and non-equilibria.

p36) Supplementary Figure 1: Plasmid relatedness.

p37) Supplementary Figure 2: Reducing the number of founders (*N*) facilitates plasmid-mediated cooperation.

p38) Supplementary information references

Appendix A: Mathematical model.

Possible genotypes

We track evolutionary dynamics at two loci: one plasmid locus, and one chromosomal locus. Individuals are haploid. We use the notation *ab* to denote the genotype of a given focal individual. At the chromosomal locus, an individual has either a 'cooperate' (*a*=2) or 'defect' (i.e. 'do not cooperate' / 'cheat'; *a*=1) allele. At the plasmid locus, an individual has either a 'cooperate' plasmid (*b=2*), a 'defect' ('do not cooperate' / 'cheat') plasmid (*b*=1), or no plasmid (*b*=0). This leads to 6 possible genotypes: 10,11,12,20,21,22. The frequency of a given genotype (*ab*), at the start of a given generation, is given by x_{ab}.

We note that 'cooperation' here means *public goods production*, and 'defection' here means *no public goods production*. We assume that 'cooperation' exhibits full dominance over 'defection', which means that, regardless of whether cooperation is encoded on a plasmid, or a chromosome, or both, it will lead to the same phenotype at the individual level. In other words, genotypes 12, 20, 21 and 22 are all producers of the public good.

Lifecycle assumptions

The lifecycle is the same as Mc Ginty *et al.* (2013), except that we include the possibility of plasmid loss. See Mc Ginty *et al.* (2013) for more information on the biological rationale behind the lifecycle assumptions. We assume an infinite population of bacteria living on an infinite number of patches (infinite island model). Generations are non-overlapping, and comprise the following lifecycle stages, which are listed in chronological order:

1. Each patch is colonised by *N* independent founder cells sampled from an infinite pool of founders.

2. Founders reproduce clonally, with perfect vertical transmission of genotypes between parent and offspring cells (no mutation; no plasmid segregation). Clonal reproduction continues until there are very large numbers of individuals in each patch.
3. Individuals pair up with another (randomly chosen) individual on their patch (individuals on a patch are shuffled randomly into pairs), and if one individual has a plasmid and the other individual doesn't, the plasmid is transferred with probability β.
4. Individuals with a cooperation allele encode a public good, at cost C_G, which generates a benefit B that is shared between all members of the patch. Individuals bearing a plasmid (either cooperative or defective) suffer a cost of plasmid carriage C_C.
5. Individuals survive according to their fitness, which is determined by the costs and benefits of public goods and the cost of plasmid carriage.
6. Surviving, plasmid-bearing individuals lose their plasmid with probability s.
7. Individuals disperse to form an infinite pool of potential founders.

We note that, in our lifecycle, clonal reproduction, plasmid transfer, and plasmid loss, occur in three distinct lifecycle stages. This is an unrealistic simplifying assumption, commonly taken in theoretical models of plasmid dynamics, to make them analytically tractable (Rankin *et al.*, 2011). Of course, in nature, the three lifecycle features (reproduction, plasmid transfer, plasmid loss) occur continuously, in tandem, but treating them as discrete lifecycle stages, as we do here, is unlikely to lead to artificial results (Rankin *et al.*, 2011; Mc Ginty *et al.*, 2013; Birch, 2014, 2017). Furthermore, the ordering of clonal reproduction, plasmid transfer, and plasmid loss, is unlikely to qualitatively affect the results. For instance, Birch showed that the particular ordering of plasmid transfer and public goods production does not qualitatively influence the course of evolution (Birch, 2014, 2017).

We construct recursions, based on our lifecycle assumptions, to describe how each genotype changes in population frequency across a generation. We take an individual-based approach to these recursions, writing them in terms of individual-level costs and benefits (in this way, our formulation deviates from *Mc Ginty et al.* (2013)). So as not to bombard the reader with too much algebra all at once, we present these recursions in three steps.

First, we write a series of equations to describe, at a high level, how a given genotype changes in frequency from the beginning of a generation to just-before the 'plasmid loss' lifecycle stage (lifecycle stage 6 listed above). The frequency of a given genotype *ab* is given by x_{ab} at the start of the generation, and by x'_{ab} just-before the 'plasmid loss' lifecycle stage (lifecycle stage 6).

Second, we close these "x_{ab} to x'_{ab}" equations, by writing all variables in terms of model parameters (N,β,C_C,C_G) . This involves deriving an expression for the number of cooperators in an individual's patch.

Third, we write a series of (closed) equations to describe how each genotype changes in frequency from just-before the 'plasmid loss' lifecycle stage (lifecycle stage 6) to the end of the generation. These equations specifically capture the evolutionary consequences of plasmid loss. The frequency of a given genotype *ab* is given by x''_{ab} at the end of the generation.

Taken together, this will give us a series of closed (dynamically sufficient) recursions, describing how genotype frequencies change over a generation (from x_{ab} to x''_{ab}), which we can iterate to see how genotype frequencies change over time and what they are at equilibrium.

x_{ab} to x'_{ab} (high-level)

First, we write high-level equations to describe how genotype frequencies change from the beginning of a generation to just-before plasmid loss (lifecycle stage 6). These equations are

"high level" (or "open") because they contain extra terms (m_A; explained below) which are not yet written explicitly in terms of model parameters (N,β,C_C,C_G).

$$x'_{22} = \Bigg(x_{22} \Bigg(\frac{1}{N} \big(1 + m_{\{22\}} B - C_G - C_C\big)$$

$$+ \frac{N-1}{N} \Big(x_{22} \big(1 + m_{\{22,22\}} B - C_G - C_C\big) + x_{21} \big(1 + m_{\{22,21\}} B - C_G - C_C\big)$$

$$+ x_{20} (1+\beta) \big(1 + m_{\{22,20\}} B - C_G - C_C\big) + x_{12} \big(1 + m_{\{22,12\}} B - C_G - C_C\big)$$

$$+ x_{11} \big(1 + m_{\{22,11\}} B - C_G - C_C\big) + x_{10} \big(1 + m_{\{22,10\}} B - C_G - C_C\big)\Big)\Bigg)$$

$$+ \ \beta \frac{N-1}{N} x_{20} x_{12} \big(1 + m_{\{20,12\}} B - C_G - C_C\big)\Bigg) / W \qquad (1)$$

$$x'_{21} = \Bigg(x_{21} \Bigg(\frac{1}{N} \big(1 + m_{\{21\}} B - C_G - C_C\big)$$

$$+ \frac{N-1}{N} \Big(x_{22} \big(1 + m_{\{21,22\}} B - C_G - C_C\big) + x_{21} \big(1 + m_{\{21,21\}} B - C_G - C_C\big)$$

$$+ x_{20} (1+\beta) \big(1 + m_{\{21,20\}} B - C_G - C_C\big) + x_{12} \big(1 + m_{\{21,12\}} B - C_G - C_C\big)$$

$$+ x_{11} \big(1 + m_{\{21,11\}} B - C_G - C_C\big) + x_{10} \big(1 + m_{\{21,10\}} B - C_G - C_C\big)\Big)\Bigg)$$

$$+ \beta \frac{N-1}{N} x_{20} x_{11} \big(1 + m_{\{20,11\}} B - C_G - C_C\big)\Bigg) / W \qquad (2)$$

$$x'_{20} = \Bigg(x_{20} \Bigg(\frac{1}{N} \big(1 + m_{\{20\}} B - C_G\big)$$

$$+ \frac{N-1}{N} \Big(x_{22} (1-\beta) \big(1 + m_{\{20,22\}} B - C_G\big)$$

$$+ x_{21} (1-\beta) \big(1 + m_{\{20,21\}} B - C_G\big) + x_{20} \big(1 + m_{\{20,20\}} B - C_G\big)$$

$$+ x_{12} (1-\beta) \big(1 + m_{\{20,12\}} B - C_G\big) + x_{11} (1-\beta) \big(1 + m_{\{20,11\}} B - C_G\big)$$

$$+ x_{10} \big(1 + m_{\{20,10\}} B - C_G\big) \Big) \Bigg) \Bigg) / W \quad (3)$$

$$x'_{12} = \Bigg(x_{12} \Bigg(\frac{1}{N} \big(1 + m_{\{12\}} B - C_G - C_C\big)$$

$$+ \frac{N-1}{N} \Big(x_{22} \big(1 + m_{\{12,22\}} B - C_G - C_C\big) + x_{21} \big(1 + m_{\{12,21\}} B - C_G - C_C\big)$$

$$+ x_{20} \big(1 + m_{\{12,20\}} B - C_G - C_C\big) + x_{12} \big(1 + m_{\{12,12\}} B - C_G - C_C\big)$$

$$+ x_{11} \big(1 + m_{\{12,11\}} B - C_G - C_C\big) + x_{10} (1+\beta) \big(1 + m_{\{12,10\}} B - C_G - C_C\big) \Big) \Bigg)$$

$$+ \beta \frac{N-1}{N} x_{22} x_{10} \big(1 + m_{\{22,10\}} B - C_G - C_C\big) \Bigg) / W \quad (4)$$

$$x'_{11} = \left(x_{11} \left(\frac{1}{N} \left(1 + m_{\{11\}} B - C_C \right) \right. \right.$$

$$+ \frac{N-1}{N} \left(x_{22} \left(1 + m_{\{11,22\}} B - C_C \right) + x_{21} \left(1 + m_{\{11,21\}} B - C_C \right) \right.$$

$$+ x_{20} \left(1 + m_{\{11,20\}} B - C_C \right) + x_{12} \left(1 + m_{\{11,12\}} B - C_C \right)$$

$$\left. \left. + x_{11} \left(1 + m_{\{11,11\}} B - C_C \right) + x_{10} (1 + \beta) \left(1 + m_{\{11,10\}} B - C_C \right) \right) \right)$$

$$\left. + \beta \frac{N-1}{N} x_{21} x_{10} \left(1 + m_{\{10,21\}} B - C_C \right) \right) / W \qquad (5)$$

$$x'_{10} = \left(x_{10} \left(\frac{1}{N} \left(1 + m_{\{10\}} B \right) \right. \right.$$

$$+ \frac{N-1}{N} \left(x_{22} (1 - \beta) \left(1 + m_{\{10,22\}} B \right) + x_{21} (1 - \beta) \left(1 + m_{\{10,21\}} B \right) \right.$$

$$+ x_{20} \left(1 + m_{\{10,20\}} B \right) + x_{12} (1 - \beta) \left(1 + m_{\{10,12\}} B \right)$$

$$\left. \left. \left. + x_{11} (1 - \beta) \left(1 + m_{\{10,11\}} B \right) + x_{10} \left(1 + m_{\{10,10\}} B \right) \right) \right) \right) / W, \qquad (6)$$

where W denotes mean population fitness, and is given by:

$$W = 1 + \left(x_{20} + x_{21} + x_{22} + x_{12} + (x_{22} + x_{12}) x_{10} \beta \frac{N-1}{N} \right) (B - C_G)$$

$$- \left((x_{11} + x_{12} + x_{21} + x_{22}) \left(1 + \beta \frac{N-1}{N} (x_{10} + x_{20}) \right) \right) C_C, \qquad (7)$$

and where m_A gives the expected proportion of the patch who are cooperators (either plasmid or chromosomal). The subscript A denotes a *set*. Specifically, A denotes the set of “known

genotypes”. The patch cooperator frequency (m_A) varies depending on the genotype of the focal individual, and who the focal individual pairs up with in the plasmid transmission lifecycle stage, as we will now explain with a few examples.

As a first example, if the focal individual has a given genotype *ab*, and it pairs up with an individual who has descended from the same founder cell as itself (this occurs with probability 1/*N*), then we can be sure that one of the original founder cells had the genotype *ab* (and gave rise to both the focal individual and its partner), but we cannot be sure about the identities of the remaining *N*-1 founder cells. The patch cooperator frequency varies depending on the genotypes of the founder cells that gave rise to the patch. Therefore, in this scenario, given that we only know the identity of one founder cell, *ab*, we write the patch cooperator frequency as $m_{\{ab\}}$ (we derive $m_{\{ab\}}$ explicitly in terms of model parameters in the next section).

As a second example, if the focal individual has a given genotype *ab*, and it pairs up with an individual who has descended from a different founder cell to itself (this occurs with probability (*N*-1)/*N*), who has the genotype *cd*, then we can be sure that one of the original founder cells had the genotype *ab* (and gave rise to the focal individual), and one of the other founder cells had the genotype *cd* (and gave rise to the focal individual’s partner). We cannot be sure about the identities of the remaining *N*-2 founder cells. This leads to patch cooperator frequency of $m_{\{ab,cd\}}$ (we derive $m_{\{ab,cd\}}$ explicitly in terms of model parameters in the next section).

Our mean fitness expression (W) makes intuitive sense. It is simply given by: post-transmission cooperator frequency $\left(x_{20} + x_{21} + x_{22} + x_{12} + (x_{22} + x_{12})x_{10}\beta\frac{N-1}{N}\right)$ multiplied by the net payoff to cooperation ($B - C_G$), added to the post-transmission plasmid frequency $\left((x_{11} + x_{12} + x_{21} + x_{22})\left(1 + \beta\frac{N-1}{N}(x_{10} + x_{20})\right)\right)$ multiplied by the cost of plasmid carriage (C_C), added to the baseline fitness (1).

We will now explain the x'_{22} equation in detail. The logic here can be used to develop the other equations, *mutatis mutandis*. The overall logic behind the equation is that it is a sum of all the different ways (probabilities) in which type-22 individuals may survive to just-before lifecycle stage 6 (which is where the equation tracks genotype frequencies to). Firstly, we focus on individuals who are derived from a founder cell who had the genotype *22*. Owing to vertical inheritance from the founder cell, these individuals also have the genotype 22. These individuals comprise the proportion x_{22} of the population, and this gives rise to the first number (multiplier) on the RHS of our x'_{22} equation: x_{22}.

A fraction of these individuals, given by 1/*N*, pair up with an individual who has descended from the same founder cell as itself (and therefore also has the 22 genotype), and as a result, no plasmid transfer occurs. This gives rise to the second number (multiplier) of 1/*N*. The third number (multiplier) is $1 + m_{\{22\}}B - C_G - C_C$, and this is the (absolute) fitness of the individuals in question. Here, $m_{\{22\}}B$ gives the amount of help received by the individuals in question, and C_G and C_C give the costs incurred by the individuals in question due to, respectively, their production of public goods and their plasmid carriage.

Dividing absolute fitness by mean population fitness gives us $x_{22}\frac{1}{N}\left(1 + m_{\{22\}}B - C_G - C_C\right)/W$, which is the proportion of the individuals in question who survive to become 22-cells at just-before lifecycle stage 6. This is one way in which type-22 individuals may reach just-before lifecycle stage 6 – it is the first term on the RHS of our x'_{22} equation.

Now, we focus on individuals who are derived from a founder cell who had the genotype *22* (proportion x_{22} of the population), and who pair up with an individual who has descended from a different founder cell as itself (occurs with probability (*N*-1)/*N*). Of these individuals, a proportion of them, given by x_{22}, are paired up with an individual who has the *22* genotype, and as a result, no plasmid transfer occurs. These individuals have a relative fitness of

$\left(1 + m_{\{22,22\}}B - C_G - C_C\right)/W$. Combining these multipliers gives the next term on the RHS of our x'_{22} equation: $x_{22}\frac{N-1}{N}\left(1 + m_{\{22,22\}}B - C_G - C_C\right)/W$.

Next, we remain focused on individuals who are derived from a founder cell who had the genotype *22* (proportion x_{22} of the population), and who pair up with an individual who has descended from a different founder cell as itself (occurs with probability (*N*-1)/*N*). Of these individuals, a proportion of them, given by x_{21}, are paired up with an individual who has the *21* genotype, and as a result, no plasmid transfer occurs. These individuals have a relative fitness of $\left(1 + m_{\{22,21\}}B - C_G - C_C\right)/W$. Combining these multipliers gives the next term on the RHS of our x'_{22} equation: $x_{22}\frac{N-1}{N}\left(1 + m_{\{22,21\}}B - C_G - C_C\right)/W$.

Next, we remain focused on individuals who are derived from a founder cell who had the genotype *22* (proportion x_{22} of the population), and who pair up with an individual who has descended from a different founder cell as itself (occurs with probability (*N*-1)/*N*). Of these individuals, a proportion of them, given by x_{20}, are paired up with an individual who has the *20* genotype (i.e. cooperative but lacks a plasmid). Of these individuals, a proportion of them, given by β, transfer their plasmid to their plasmid-free partner, establishing new 22-genotype individuals by horizontal transfer. This leads to the $1 + \beta$ multiplier. The relative fitness of the 22-genotype individuals involved in the 22 & 20 pairs is given by $\left(1 + m_{\{22,20\}}B - C_G - C_C\right)/W$. Combining these multipliers gives the next term on the RHS of our x'_{22} equation: $x_{22}\frac{N-1}{N}(1+\beta)\left(1 + m_{\{22,20\}}B - C_G - C_C\right)/W$.

The next three terms are arrived at through similar reasoning to the previous three, so we don't go over these explicitly. This takes us to our final term. To explain this term, we need to focus on pairings in the population that are between type-20 and type-12 individuals. Such pairings comprise a fraction $\frac{N-1}{N}x_{20}x_{12}$ of all parings in the population. In a proportion β of

these pairings, a plasmid is transferred from the type-12 individual to the type-20 individual, to create a type-22 individual. The relative fitness of the resulting type-22 individuals is $\left(1 + m_{\{20,12\}}B - C_G - C_C\right)/W$. Combining these multipliers gives $\beta\frac{N-1}{N}x_{20}x_{12}\left(1 + m_{\{20,12\}}B - C_G - C_C\right)/W$, which is the final way in which type-22 individuals may reach just-before lifecycle stage 6 – it is the final term on the RHS of our x'_{22} equation.

<u>Closing our x_{ab} to x'_{ab} equations by deriving m_A explicitly</u>

We now derive the patch cooperator frequency, m_A, explicitly in terms of model parameters. The patch cooperator frequency varies depending on what we 'know' about the genotypes of founder cells. From the perspective of a given focal individual, who has descended from an *ab* founder cell, we know (trivially) the identity of one of the cells who founded the patch – specifically, we know that one founder cell had the genotype *ab*. If our focal individual pairs up with another individual who has descended from the same founder cell as itself, we gain no further information about the identities of the *N* founder cells. In this case, there are *N*-1 'unknown' founder cells.

However, if our focal cell pairs up with an individual who has descended from a different founder cell to itself (and has genotype *cd,* which may or may not be the same as *ab*), we gain further information about the identities of the *N* founder cells. Specifically, in this case, there are two 'known' founder cells, which are type-*ab* and type-*cd*, and N-2 'unknown' founder cells.

Therefore, in a given scenario, there are either *N*-1 unknown founder cells (1 known), or *N*-2 unknown founder cells (2 known). The task now is to calculate, for each scenario, the (expected) genotypes of the 'unknown' founder cells. Then, using this information about the

genotypes of the founder cells, we can calculate, for each scenario, the resulting (expected) patch cooperator frequency.

To reiterate, we take A as the set of known founder cells. Therefore, to take an arbitrary example, for a focal individual who has descended from a type-20 founder cell, and who pairs with an individual who has descended from a type-12 founder cell, our set A would equal *{20,12}*.

We now let *i* be the number of chromosomal cooperators (genotypes 20 & 21) in the set of known founder genotypes (A). We let *j* be the number of plasmid cooperators (genotypes 22 & 12) in the set of known founder genotypes (A). We let k be the number of plasmid-free defectors (genotype 10) in the set of known founder genotypes (A). We let *l* be the number of double-locus-defectors (genotype 11) in the set of known founder genotypes (A). We let $K = i + j + k + l$ be the total number of known founders, meaning there are $N - K$ unknown founders.

Now, we let $p_K(n_{20}, n_{21}, n_{22}, n_{10}, n_{11}, n_{12})$ denote the probability that, in addition to our set (A) of K 'known' individuals, a patch is founded by exactly n_{20} type-20 individuals, n_{21} type-21 individuals, n_{22} type-22 individuals, n_{10} type-10 individuals, n_{11} type-11 individuals, and n_{12} type-12 individuals. We can then write this probability $p_K(n_{20}, n_{21}, n_{22}, n_{10}, n_{11}, n_{12})$ as the probability mass function of a multinomial random variable, as follows:

$$p_K(n_{20}, n_{21}, n_{22}, n_{10}, n_{11}, n_{12})$$

$$= \Pr(N_{20} = n_{20}, N_{21} = n_{21}, N_{22} = n_{22}, N_{20} = n_{20}, N_{21} = n_{21}, N_{22} = n_{22})$$

$$= \begin{cases} \frac{K!}{n_{20}! n_{21}! n_{22}! n_{10}! n_{11}! n_{12}!} x_{20}^{n_{20}} x_{21}^{n_{21}} x_{22}^{n_{22}} x_{10}^{n_{10}} x_{11}^{n_{11}} x_{12}^{n_{12}}, & \text{if } \mathrm{n}_{20} + n_{21} + n_{22} + n_{10} + n_{11} + n_{12} = K, \\ 0, & \text{otherwise.} \end{cases}$$

(8)

We condition this on the numbers of the various types of 'known' founders, and divide through by N. This gives us an expression for the patch cooperator frequency (dividing though by N ensures that cooperator frequency is given relative to patch size, i.e. as a *proportion* of the patch):

$$m_A = \frac{1}{N} \sum_{n_{10}=0}^{N-K} \sum_{n_{11}=0}^{N-K} \sum_{n_{12}=0}^{N-K} \sum_{n_{20}=0}^{N-K} \sum_{n_{21}=0}^{N-K} \sum_{n_{22}=0}^{N-K} p_K(n_{20}, n_{21}, n_{22}, n_{10}, n_{11}, n_{12}) \Big(i + j + n_{20} + n_{21} + n_{22} + n_{12} + \beta(n_{10} + k) \frac{j + n_{22} + n_{12}}{N} \Big). \quad (9)$$

We can write this in terms of expectations and covariances as:

$$m_A = \frac{1}{N} \Bigg(i + j + \frac{jk\beta}{N} + \frac{j\beta}{N} E[N_{10}] + E[N_{12}] \left(1 + \frac{k\beta}{N}\right) + \frac{\beta}{N} (\mathrm{Cov}(N_{10}, N_{12}) + E[N_{10}]E[N_{12}]) + E[N_{20}] + E[N_{21}] + E[N_{22}] \left(1 + \frac{k\beta}{N}\right) + \frac{\beta}{N} (\mathrm{Cov}(N_{10}, N_{22}) + E[N_{10}]E[N_{22}]) \Bigg). \quad (10)$$

We can evaluate this using $E[N_{10}] = x_{10}(N-K), E[N_{12}] = x_{12}(N-K), E[N_{20}] = x_{20}(N-K), E[N_{21}] = x_{21}(N-K), E[N_{22}] = x_{22}(N-K), \mathrm{Cov}(N_{10}, N_{12}) = -(N-K)x_{10}x_{12}, \mathrm{Cov}(N_{10}, N_{22}) = -(N-K)x_{10}x_{22}$. We obtain:

$$m_A = \frac{Q}{N^2}, \tag{11}$$

where

$$\begin{aligned} Q = {} & N(i+j) + \beta jk \\ & + (K-N)\Big(\beta x_{10}\big(-j + x_{12}(K-N+1) + x_{22}(K-N+1)\big) \\ & + x_{12}\big(-(\beta k + N)\big) - x_{22}(\beta k + N) - N(x_{20} + x_{21})\Big). \end{aligned} \tag{12}$$

And with this, we have a general-form expression, which we can use to obtain the specific m_A terms in the above equations. To obtain the specific m_A term for a given set A, we simply substitute in the i,j and k values associated with the set. For instance, for the set {20,11}, we have i=1,j=0,k=0,l=1, which leads to the following patch cooperator frequency:

$$m_{\{20,11\}} = \frac{Q|_{i=1,j=0,k=0,l=1}}{N^2}$$

$$= \frac{(N-2)(x_{12}(\beta(N-3)x_{10} + N) + x_{22}(\beta(N-3)x_{10} + N) + N(x_{20} + x_{21})) + N}{N^2}. \tag{13}$$

x'_{ab} to x''_{ab}

Having now obtained "closed" equations to describe how each genotype changes in frequency from the start of the generation to just-before the 'plasmid loss' lifecycle stage (lifecycle stage 6) ($x_{ab}$to x'_{ab}), we now write equations to describe how each genotype changes in frequency from just-before the 'plasmid loss' lifecycle stage (lifecycle stage 6) to the end of the generation (x'_{ab} to x''_{ab}). These equations specifically capture the evolutionary consequences of plasmid loss.

We take *z* to be 1 or 2 ($z \in \{1,2\}$). This means that *az* refers to a given plasmid-carrying genotype (i.e., it includes the genotypes $a1$ and *a2*), and *a0* refers to a given plasmid-free genotype. The frequency of a given plasmid-carrying genotype at the end of the generation (x''_{az}), and the frequency of a given plasmid-free genotype at the end of the generation (x''_{a0}), can then be written:

$$x''_{az} = (1 - s)x'_{az}, \tag{14}$$

$$x''_{a0} = x'_{a0} + s \sum_{z=1}^{2} x'_{az}\,, \tag{15}$$

where *s* denotes the probability that a given plasmid-bearing individual loses its plasmid. Note that Equation 14 applies to genotypes with cooperative plasmids (*a2*) as well as genotypes with defector plasmids (*a1*).

Now, we can take our closed equations describing how genotype frequencies change from the start of the generation to just-before plasmid loss (x_{ab} to x'_{ab}), alongside our equations describing how genotype frequencies change from just-before plasmid loss to the end of the

generation (x'_{ab} to x''_{ab}). This gives us a series of dynamically sufficient *recursions*, which describe how genotype frequencies change over an entire generation (from x_{ab} to x''_{ab}).

We note that, in the special case where plasmid loss is absent (*s*=0), And where only the 10 genotype (chromosomal defection) and the 12 genotype (plasmid-mediated cooperation) are present in the population, such that $x_{10} + x_{12} = 1, x_{11} = 0, x_{20} = 0, x_{21} = 0, x_{22} = 0$, our recursions simplify to capture the scenario modelled by *Mc Ginty et al.* (Mc Ginty *et al.*, 2013).

Solving the model

Having obtained dynamically sufficient recursions, we can now solve the model, to see what genotypes evolve. To do so, we assume that, initially, cooperation is absent from the population, and there are no plasmids. We then introduce the remaining five genotypes from rarity. To be specific, we take the following initial genotype frequencies: $x_{10} = 0.999, x_{11} = 0.0002, x_{12} = 0.0002, x_{20} = 0.0002, x_{21} = 0.0002, x_{22} = 0.0002$. We then track the evolutionary process, by numerically iterating our recursions over successive generations. We continue tracking evolution for 100,000 generations, at which point, genotype frequencies are changing negligibly, meaning evolutionary equilibrium has been reached (either approximately or exactly).

Appendix B: Derivation of plasmids relatedness in terms of model parameters.

This appendix derives a fully explicit expression for plasmid relatedness in terms of model parameters. Doing so reveals that the general relatedness expression derived by Mc Ginty *et al.* (2013) is incorrect, though it is approximately correct in the special cases considered by Mc Ginty *et al.* (2013). Note that the population genetic recursions derived in Appendix A are fully complete. We derive relatedness in this appendix for interpreting the model outlined in Appendix A, not formulating it. Readers not interested in the mathematical details can skip this appendix, and refer to Figure 1B–D to see how the plasmid relatedness expression behaves (derived as Equation 29 below).

<u>Notation</u>

First, we recap some model assumptions, and define some new variables. Each patch is colonised by exactly N founders. Each founder $i = 1,...,N$ proliferates equally, giving rise to a lineage $i = 1,...,N$, where each lineage comprises the same number of descendants.

Let I_i denote an indicator variable that equals 1 if founder $i = 1,...,N$ carries a cooperator plasmid, and 0 otherwise. Each founder has a p_C chance of having a cooperator plasmid, where p_C is the population frequency of the cooperator plasmid (p_C=x_{12}+x_{22}). Let I_i' denote the frequency of the cooperator plasmid amongst descendants of founder i, measured at the point of public goods production (meaning horizontal plasmid transfer has occurred).

Let J_i denote an indicator variable that equals 1 if founder $i = 1,...,n$ carries a defector plasmid, and 0 otherwise. Each founder has a p_D chance of having a defector plasmid, where p_D is the population frequency of the defector plasmid (p_C=x_{11}+x_{21}). The indicator variables I_i

and J_i are related such that: if $I_i = 1$, then $J_i = 0$; if $J_i = 1$, then $I_i = 0$; if $I_i = 0$, then $J_i = 0$ or 1; if $J_i = 0$, then $I_i = 0$ or 1. These relations are simply saying that, if an individual (i) has a plasmid of a given type (cooperator or defector), then it cannot simultaneously have a plasmid of the other type (in accordance with our model assumptions).

Let $P = \left(\frac{1}{N}\right) \sum_{i=1}^{N} I_i$ denote the proportion of founders on a given patch who carry the cooperator plasmid. Let P' denote the patch frequency of the cooperator plasmid at the point of public goods production (i.e., after horizontal plasmid transmission). It is also convenient to define $S_N = NP$ as the sum of founders on a given patch who carry the cooperator plasmid, with $S_N' = NP'$ denoting the effective number of founding lineages who carry the cooperator plasmid at the point of public goods production (i.e., after horizontal plasmid transmission).

General definition of relatedness

Relatedness defined at a plasmid locus is given by the following formula (Grafen, 1985; Frank, 1998; Gardner *et al.*, 2011; Rousset, 2015). It measures genetic similarity at the plasmid locus:

$$R_{plas} = \frac{\mathrm{Cov}(P_N', I_1')}{\mathrm{Var}(I_1')}. \quad (16)$$

This is a "whole-group" relatedness and is interpretable as a regression (Pepper, 2000). We will now derive an explicit version of R_{plas}, expressed in terms of model parameters. To do so, we will gradually re-write our relatedness expression, evaluating higher level factors in terms of lower level ones, until it is expressed completely in terms of model parameters.

It is convenient to write our relatedness expression in a slightly different form. Specifically, we can write covariance and variance in terms of expectations (using standard identities). We can also recognise that the expected proportion of the cooperator plasmid on a

patch, at a given point in time, will be equal to the expected proportion of the cooperator plasmid in an individual drawn from the patch, meaning $E[P'] = E[I_1']$. Finally, we can substitute $P' = \left(\frac{1}{N}\right) S_N'$. This allows us to write Equation 16 as:

$$R_{plas} = \frac{\left(\frac{1}{N}\right) E[S_N' I_1'] - E[I_1']^2}{E\left[{I_1'}^2\right] - E[I_1']^2}. \quad (17)$$

Next, we can substitute in explicit expressions for I_1', ${I_1'}^2$ and $S_N' I_1'$.

<u>Deriving I_1'</u>

The frequency of the cooperator plasmid amongst descendants of founder 1 (I_1') can be written explicitly as:

$$I_1' = I_1 + \frac{\beta}{N}(1 - I_1 - J_1) \sum_{i=2}^{N} I_i \, . \quad (18)$$

To interpret this expression, note that, if $I_1 = 1$, meaning the founder (1) had the cooperator plasmid, all descendants of founder 1 will inherit the cooperator plasmid by vertical transmission, leading to $I_1' = I_1$ (the second term is eliminated because $1 - I_1 - J_1 = 0$ whenever $I_1 = 1$, owing to I / J relationship described above). Conversely, if $J_1 = 1$, meaning the founder (1) had the defector plasmid, all descendants of founder 1 will inherit the defector plasmid by vertical transmission, leading to $I_1' = 0$ (the first and second terms are eliminated because $I_1 = 0$ and $1 - I_1 - J_1 = 0$ whenever $J_1 = 1$, owing to I / J relationship described above). Conversely, if $I_1 = 0$ & $J_1 = 0$, meaning the founder (1) had no plasmid, the proportion $\left(\frac{\beta}{N}\right) \sum_{i=2}^{N} I_i$ of founder 1 descendants will receive the plasmid horizontally from descendants of founders who had the cooperator plasmid.

Deriving ${I'_1}^2$

The square of the frequency of the cooperator plasmid amongst descendants of founder 1 (I'_1) can be written explicitly as: ${I'_1}^2 = \left(I_1 + \frac{\beta}{N}(1 - I_1 - J_1)\sum_{i=2}^{N} I_i\right)^2$ (the right hand side of this equation is obtained simply by squaring the right hand side of Equation 18). We can expand this as: ${I'_1}^2 = \left({I_1}^2 + \left(\frac{\beta}{N}\right)^2 (1 - I_1 - J_1)^2 (\sum_{i=2}^{N} I_i)^2 + 2I_1\left(\frac{\beta}{N}(1 - I_1 - J_1)\sum_{i=2}^{N} I_i\right)\right)^2$. Noting that $I_1(1 - I_1 - J_1) = 0$ (owing to I / J relationship described above), this simplifies to:

$$ {I'_1}^2 = {I_1}^2 + \left(\frac{\beta}{N}\right)^2 (1 - I_1 - J_1)^2 \left(\sum\nolimits_{i=2}^{N} I_i\right)^2 . \qquad (19) $$

Deriving $S'_N I'_1$

The effective number of founding lineages who have the plasmid at the point of public goods production (S'_n) can be written explicitly as: $S'_N = I_1 + \frac{\beta}{N}(1 - I_1 - J_1)\sum_{i=2}^{N} I_i + \sum_{i=2}^{N} I_i + \frac{\beta}{N}\sum_{i=2}^{N}(1 - I_i - J_i)\left(\sum_{j \neq i,1}^{N} I_j + I_1\right)$. The product of the frequency of the cooperator plasmid amongst descendants of founder 1 and the effective number of founding lineages who have the plasmid at the point of public goods production ($S'_n I'_1$) can therefore be written explicitly as: $S'_N I'_1 = \left(I_1 + \frac{\beta}{N}(1 - I_1 - J_1)\sum_{i=2}^{N} I_i\right)\left(I_1 + \frac{\beta}{N}(1 - I_1 - J_1)\sum_{i=2}^{N} I_i + \sum_{i=2}^{N} I_i + \frac{\beta}{N}\sum_{i=2}^{N}(1 - I_i - J_i)\left(\sum_{j \neq i,1}^{N} I_j + I_1\right)\right)$. This can be expanded and simplified using Equation 18 to obtain:

$$S_N' I_1' = {I_1'}^2 + I_1 \sum_{i=2}^{N} I_i + I_1 \frac{\beta}{N} \sum_{i=2}^{N} (1 - I_i - J_i) \left(\sum_{j \neq i,1}^{N} I_j + I_1 \right)$$
$$+ \frac{\beta}{N} (1 - I_1 - J_1) \left(\sum_{i=2}^{N} I_i \right)^2$$
$$+ \left(\frac{\beta}{N}\right)^2 (1 - I_1 - J_1) \sum_{i=2}^{N} I_i \left(\sum_{i=2}^{N} (1 - I_i - J_i) \left(\sum_{j \neq i,1}^{N} I_j + I_1 \right) \right). \quad (20)$$

It is convenient to write the fifth term of the right hand side of Equation 20 slightly differently. First, we expand it: $\left(\frac{\beta}{n}\right)^2 (1 - I_1 - J_1) \sum_{i=2}^{N} I_i \left(\sum_{i=2}^{N} (1 - I_i - J_i) \left(\sum_{j \neq i,1}^{N} I_j + I_1 \right) \right) = \left(\frac{\beta}{N}\right)^2 \sum_{i=2}^{N} I_i \left(\sum_{i=2}^{N} (1 - I_i - J_i) \left(\sum_{j \neq i,1}^{N} I_j (1 - I_1 - J_1) + I_1 (1 - I_1 - J_1) \right) \right)$. Next, by taking the *i* terms out of the following sum and noting that $I_i (1 - I_i - J_i) = 0$ (owing to the I / J relations stated above), we can re-write $I_i \sum_{i=2}^{N} (1 - I_i - J_i) = I_i (1 - I_i - J_i) + I_i \sum_{k \neq 1,i}^{N} (1 - I_k - J_k) = I_i \sum_{k \neq 1,i}^{N} (1 - I_k - J_k)$, which simplifies the overall term to: $\left(\frac{\beta}{N}\right)^2 (1 - I_1 - J_1) \sum_{i=2}^{N} I_i \left(\sum_{i=2}^{N} (1 - I_i - J_i) \left(\sum_{j \neq i,1}^{N} I_j + I_1 \right) \right) = \left(\frac{\beta}{N}\right)^2 \sum_{i=2}^{N} I_i \left(\sum_{k \neq 1,i}^{N} (1 - I_k - J_k) \left(\sum_{j \neq i,1}^{N} I_j (1 - I_1 - J_1) + I_1 (1 - I_1 - J_1) \right) \right)$. We can expand $\sum_{j \neq i,1}^{N} I_j (1 - I_1 - J_1) = \sum_{j \neq i,1,k}^{N} I_j (1 - I_1 - J_1) + I_k (1 - I_1 - J_1)$, which means the overall term is given by: $\left(\frac{\beta}{N}\right)^2 \sum_{i=2}^{N} I_i \left(\sum_{k \neq 1,i}^{N} (1 - I_k - J_k) \left(\sum_{j \neq i,1,k}^{N} I_j (1 - I_1 - J_1) + I_k (1 - I_1 - J_1) + I_1 (1 - I_1 - J_1) \right) \right)$. By expansion this becomes: $\left(\frac{\beta}{N}\right)^2 \sum_{i=2}^{N} I_i \left(\sum_{k \neq 1,i}^{N} \left(\sum_{j \neq i,1,k}^{N} I_j (1 - I_1 - J_1)(1 - I_k - J_k) + I_k (1 - I_1 - J_1)(1 - I_k - J_k) + I_1 (1 - I_1 - J_1)(1 - I_k - J_k) \right) \right)$. By further expansion this becomes: $\left(\frac{\beta}{n}\right)^2 \sum_{i=2}^{N} \left(\sum_{k \neq 1,i}^{N} \left(\sum_{j \neq i,1,k}^{N} I_i I_j (1 - I_1 - J_1)(1 - I_k - J_k) + I_i I_k (1 - I_1 - J_1)(1 - I_k - J_k) + I_i I_1 (1 - I_1 - J_1)(1 - I_k - J_k) \right) \right)$. By noting that $I_1 (1 - I_1 - J_1) = 0$, we

can obtain the full simplified term as: $\left(\frac{\beta}{N}\right)^2 \sum_{i=2}^{N} \left(\sum_{k\neq 1,i}^{N} \left(\sum_{j\neq i,1,k}^{N} I_i I_j\, (1 - I_1 - J_1)(1 - I_k - J_k) + I_i I_k (1 - I_1 - J_1)(1 - I_k - J_k)\right)\right)$. This gives us the following simplified expression for $S'_N I'_1$:

$$S'_N I'_1 = {I'_1}^2 + I_1 \sum_{i=2}^{N} I_i + I_1 \frac{\beta}{N} \sum_{i=2}^{N} (1 - I_i - J_i) \left(\sum_{j\neq i,1}^{N} I_j + I_1\right) + \frac{\beta}{N}(1 - I_1 - J_1)\left(\sum_{i=2}^{N} I_i\right)^2 + \left(\frac{\beta}{N}\right)^2 \sum_{i=2}^{N} \left(\sum_{k\neq 1,i}^{N} \left(\sum_{j\neq i,1,k}^{N} I_i I_j\, (1 - I_1 - J_1)(1 - I_k - J_k) + I_i I_k (1 - I_1 - J_1)(1 - I_k - J_k)\right)\right). \tag{21}$$

We can use our explicit expressions for I'_1, ${I'_1}^2$ and $S'_n I'_1$ to derive explicit expressions for the expectation terms that feature in Equation 17 (our relatedness expression): $E[I'_1]$, $E\left[{I'_1}^2\right]$ and $E[S'_n I'_1]$.

<u>Deriving $E[I'_1]$</u>

First, we will derive $E[I'_1]$, which denotes the expected proportion of lineage 1 descendants who have the cooperator plasmid, where the expectation is taken over all patches. By taking an expectation over both the left- and right-hand sides of Equation 18, we obtain: $E[I'_1] = E\left[I_1 + \frac{\beta}{N}(1 - I_1 - J_1) \sum_{i=2}^{N} I_i\right]$. Given that the expected value of the sum of random variables is equal to the sum of their expectations, we can write this equivalently as: $E[I'_1] = E[I_1] + \frac{\beta}{N} E[(1 - I_1 - J_1) \sum_{i=2}^{N} I_i]$. Given that I_1 and $\sum_{i=2}^{N} I_i$ are statistically independent (the 1 subscript doesn't feature in the sum over *i*), we can write this equivalently as: $E[I'_1] = E[I_1] +$

$\frac{\beta}{N}E[(1-I_1-J_1)]E[\sum_{i=2}^{N} I_i]$. Noting that I_1 and $1-I_1-J_1$ follow Bernoulli distributions with respective parameters ("probabilities of success") p_C and $1-p_C-p_D$, and that the mean of a Bernoulli distribution is given by its parameter, we obtain $E[I_1]=p_C$ and $E[(1-I_1-J_1)] = 1-p_C-p_D$. Noting that $\sum_{i=2}^{N} I_i$ follows a binomial distribution with parameters *success* = p_C ("probability of success") and *trials* = N – 1 ("number of trials"), and that the mean of a binomial distribution is given by *trials***success*, we obtain $E[\sum_{i=2}^{N} I_i]=(N-1)p_C$. Putting this together, we obtain:

$$E[I_1'] = p_C + \left(\frac{\beta}{N}(1-p_C-p_D)\right)(N-1)p_C. \quad (22)$$

Deriving $E[I_1']$

Next, we will derive $E\left[{I_1'}^2\right]$, which denotes the square of the expected proportion of lineage 1 descendants who have the cooperator plasmid, where the expectation is taken over all patches. By taking an expectation over both the left- and right-hand sides of Equation 19, we obtain: $E\left[{I_1'}^2\right] = E\left[{I_1}^2 + \left(\frac{\beta}{N}\right)^2(1-I_1-J_1)^2(\sum_{i=2}^{N} I_i)^2\right]$. Given that the expected value of the sum of random variables is equal to the sum of their expectations, and that $(1-I_1-J_1)^2$ and $(\sum_{i=2}^{N} I_i)^2$ are statistically independent, this becomes: $E\left[{I_1'}^2\right] = E\left[{I_1}^2\right] + \left(\frac{\beta}{N}\right)^2 E[(1-I_1-J_1)^2]E\left[\sum_{i=2}^{N} {I_i}^2\right]$.

Noting again that I_1 follows a Bernoulli distribution with parameter *success* = p_C, and that the mean and variance of a Bernoulli distribution are respectively given by *success* and *success**(1–*success*), we can write $\mathrm{Var}(I_1) = E\left[{I_1}^2\right] - E[I_1]^2 = p_C(1-p_C)$ and $E[I_1]^2 = {p_C}^2$, which can be rearranged and simplified to obtain $E\left[{I_1}^2\right] = p_C$. Also noting that $(1-I_1-J_1)$ follows a Bernoulli distribution with parameter $success = 1-p_C-p_D$, we

obtain $E[(1-I_1-J_1)^2]=1-p_C-p_D$, *mutatis mutandis*. Again, noting that $\sum_{i=2}^{N} I_i$ follows a binomial distribution with parameters *success* = p_C and *trials* = $N-1$, and that the mean and variance of a binomial distribution are respectively given by *trials***success* and *trials***success**(1–*success*), we can write $\mathrm{Var}(\sum_{i=2}^{N} I_i)=E\left[\sum_{i=2}^{N} {I_i}^2\right]-E[\sum_{i=2}^{N} I_i]^2=(N-1)p_C(1-p_C)$ and $E[\sum_{i=2}^{N} I_i]^2=\left((N-1)p_C\right)^2$, which can be rearranged to obtain $E\left[\sum_{i=2}^{N} {I_i}^2\right]=(N-1)p_C(1-p_C)+(N-1)^2{p_C}^2$. Putting this together, we obtain:

$$E\left[{I_1'}^2\right]=p_C+\left(\frac{\beta}{N}\right)^2(1-p_C-p_D)\left((N-1)p_C(1-p_C)+(N-1)^2{p_C}^2\right). \qquad (23)$$

<u>Deriving $E[S_N'I_1']$</u>

Next, we will derive $E[S_N'I_1']$, which denotes the expected product of the frequency of the cooperator plasmid amongst descendants of founder 1 and the effective number of founding lineages who have the plasmid at the point of public goods production. By taking an expectation over both the left- and right-hand sides of Equation 21, we obtain: $E[S_N'I_1']=E\Big[{I_1'}^2+I_1\sum_{i=2}^{N} I_i+I_1\frac{\beta}{N}\sum_{i=2}^{N}(1-I_i-J_i)\left(\sum_{j\neq i,1}^{N} I_j+I_1\right)+\frac{\beta}{N}(1-I_1-J_1)(\sum_{i=2}^{N} I_i)^2+\left(\frac{\beta}{N}\right)^2\sum_{i=2}^{N}\left(\sum_{k\neq 1,i}^{N}\left(\sum_{j\neq i,1,k}^{N} I_iI_j(1-I_1-J_1)(1-I_k-J_k)+I_iI_k(1-I_1-J_1)(1-I_k-J_k)\right)\right)\Big]$. Given that the expected value of the sum of random variables is equal to the sum of their expectations, and that the expectation of the product of statistically independent random variables is equal to the product of their expectations, we can write this equivalently as:

$$E[S_N' I_1'] = E\left[{I_1'}^2\right] + E[I_1]E\left[\sum_{i=2}^{N} I_i\right] + \frac{\beta}{N}E\left[I_1\sum_{i=2}^{N}(1 - I_i - J_i)\left(\sum_{j\neq i,1}^{N} I_j + I_1\right)\right]$$

$$+\frac{\beta}{N}E[(1 - I_1 - J_1)]E\left[\left(\sum_{i=2}^{N} I_i\right)^2\right]$$

$$+\left(\frac{\beta}{N}\right)^2 E\left[\sum_{i=2}^{N}\left(\sum_{k\neq 1,i}^{N}\left(\sum_{j\neq i,1,k}^{N} I_i I_j\,(1 - I_1 - J_1)(1 - I_k - J_k)\right.\right.\right.$$

$$\left.\left.\left.+ I_i I_k(1 - I_1 - J_1)(1 - I_k - J_k)\right)\right)\right]. \quad (24)$$

We will now evaluate the five terms comprising the right-hand side of Equation 24. The first term, $E\left[{I_1'}^2\right]$, has already been evaluated (Equation 23).

We can use properties of the binomial distribution to write the second term as:

$$E[I_1]E\left[\sum_{i=2}^{N} I_i\right] = (N-1){p_C}^2. \quad (25)$$

The third term can be expanded to obtain: $\frac{\beta}{N}E\left[I_1\sum_{i=2}^{N}(1 - I_i - J_i)\left(\sum_{j\neq i,1}^{N} I_j + I_1\right)\right] = \frac{\beta}{n}E\left[\sum_{i=2}^{N}(1 - I_i - J_i)\left(\sum_{j\neq i,1}^{N} I_j\, I_1 + {I_1}^2\right)\right]$. Statistical independence means that we can write this as: $\frac{\beta}{N}E[\sum_{i=2}^{N}(1 - I_i - J_i)]E\left[\left(\sum_{j\neq i,1}^{N} I_j\, I_1 + {I_1}^2\right)\right]$. Writing the expectation of a sum of random variables as the sum of their expectations, this becomes: $\frac{\beta}{n}E[\sum_{i=2}^{N}(1 - I_i - J_i)]\left(\sum_{j\neq i,1}^{N} E\left[I_j I_1\right] + E\left[{I_1}^2\right]\right)$. By statistical independence, this then becomes: $\frac{\beta}{N}E[\sum_{i=2}^{N}(1 - I_i - J_i)]\left(\sum_{j\neq i,1}^{N} E\left[I_j\right]E[I_1] + E\left[{I_1}^2\right]\right)$. Finally, using properties of the binomial distribution, and simplifying, we arrive at an explicit expression for our third term:

$$\frac{\beta}{N}E\left[I_1\sum_{i=2}^{n}(1-I_i-J_i)\left(\sum_{j\neq i,1}^{N}I_j+I_1\right)\right]$$

$$=\frac{\beta}{N}(n-1)(1-p_C-p_D)\big((N-2){p_C}^2+p_C\big). \tag{26}$$

We can use properties of the binomial distribution to write the fourth term as:

$$\frac{\beta}{N}E[(1-I_1-J_1)]E\left[\left(\sum_{i=2}^{N}I_i\right)^2\right]$$

$$=\frac{\beta}{N}(1-p_C-p_D)\big((N-1)p_C(1-p_C)+(N-1)^2{p_C}^2\big). \tag{27}$$

We can use properties of the binomial distribution to write the fifth term as:

$$\left(\frac{\beta}{N}\right)^2E\left[\sum_{i=2}^{N}\left(\sum_{k\neq 1,i}^{N}\left(\sum_{j\neq i,1,k}^{N}I_iI_j\,(1-I_1-J_1)(1-I_k-J_k)\right.\right.\right.$$

$$\left.\left.\left.+\,I_iI_k(1-I_1-J_1)(1-I_k-J_k)\right)\right)\right]$$

$$=\left(\frac{\beta}{N}\right)^2(N$$

$$-\,1)\Big((N-2)\big((N-3){p_C}^2(1-p_C-p_D)^2+p_c(1-p_C-p_D)^2\big)\Big). \tag{28}$$

<u>Full relatedness expression in terms of model parameters</u>

By evaluating the terms on the right-hand side of Equation 17 using Equations 22–28, we arrive at the following fully explicit relatedness expression:

$$R_{plas} = \Bigg(\bigg(p_C - \frac{\beta\ p_C\,(n - 1)(p_C + p_D - 1)}{N}\bigg)^2$$

$$- \bigg(p_C + p_C{}^2\ (N - 1)$$

$$- \frac{2\,\beta\ p_C\,(N - 1)(p_C + p_D - 1)(N\,p_C - 2\,p_C + 1)}{N}$$

$$- \frac{\big(\beta^2\,p_C\,(N - 1)(p_C + p_D - 1)(N\,p_C - 2\,p_C + 1)\big)}{N^2}$$

$$+ \frac{\big(\beta^2\,p_C\,(N - 1)(N - 2)\,(p_C + p_D - 1)^2\,(N\,p_C - 3\,p_C + 1)\big)}{N^2}\bigg)/N\Bigg)$$

$$/\Bigg(\bigg(p_C - \frac{\beta\ p_C\,(n - 1)(p_C + p_D - 1)}{N}\bigg)^2 - p_C$$

$$+ \big(\beta^2\,p_C\,(N - 1)(p_C + p_D - 1)\,(N\,p_C - 2\,p_C + 1)\big)/N^2\Bigg). \qquad (29)$$

We check, using simulation, that our relatedness expression (Equation 29) is correct. To do so, first we assume specific values for p_C, p_D, N & β. We obtain a simulated combined I+J array by drawing N values from a binomial distribution centred at p_C+p_D. We obtain a simulated *plasmid cooperativeness* array by drawing N values from a binomial distribution centred at p_C/(p_C+p_D). We obtain a simulated I array by multiplying our I+J array by our *plasmid cooperativeness* array. We obtain a simulated J array analogously, by multiplying our I+J array by 1–*plasmid cooperativeness*. We obtain a simulated I' array by manipulating our I array using the following relation: $I_i' = I_i + \frac{\beta}{N}(1 - I_i - J_i)\sum_{j\neq i}^{N} I_j$. We obtain I_1' by taking the first entry of this I_i' array, and we obtain P_N' by summing all entries in this I_i' array. We repeat this process many times to obtain many I_1' & P_N' combinations. Each I_1' & P_N' combination can be thought as belonging to a different patch in the population. Next, we calculate $\mathrm{Cov}(P_N', I_1')$ and $\mathrm{Var}(I_1')$,

which (using Equation 16) allows us to obtain a simulated estimate for relatedness. We find that the simulated relatedness value equals the analytical relatedness value obtained by substituting our explicit values for p_C, p_D, N & β into Equation 29. Furthermore, we find that the simulated and analytical relatedness values are equal across the full sweep of combinations of p_C, p_D, N & β values. The code for this is provided in a supplementary GitHub repository (Scott *et al.*, 2023).

The relatedness expression given in the Supplementary Information of Mc Ginty *et al.* (2013) is incorrect in general, but accurate in the special cases considered in the main text of Mc Ginty *et al.* (2013).

By taking our plasmid relatedness expression (Equation 29) and setting p_D=0, then simplifying, we obtain relatedness at the plasmid locus for the special case where all plasmids are cooperative. This was the case considered by Mc Ginty *et al.* (2013). We obtain:

$$R|_{p_D=0} = -\Bigg(\Bigg(p_C + p_C{}^2(N-1) - \frac{\big(2\beta p_C(N-1)(p_C-1)(Np_C-2p_C+1)\big)}{N}$$

$$- \frac{\big(\beta^2 p_C(N-1)(p_C-1)(Np_C-2p_C+1)\big)}{N^2}$$

$$+ \frac{\big(\beta^2 p_C(N-1)(N-2)(p_C-1)^2(Np_C-3p_C+1)\big)}{N^2}\Bigg)/N$$

$$- \left(p_C - \frac{\big(\beta p_C(N-1)(p_C-1)\big)}{N}\right)^2\Bigg)$$

$$/\Bigg(\left(p_C - \frac{\big(\beta p_C(N-1)(p_C-1)\big)}{N}\right)^2 - p_C$$

$$+ \frac{\big(\beta^2 p_C(N-1)(p_C-1)(Np_C-2p_C+1)\big)}{N^2}\Bigg). \qquad (30)$$

This should match the relatedness expression obtained Mc Ginty *et al.* (2013), but it doesn't. Mc Ginty *et al.*'s (2013) relatedness expression is given by:

$$R_{McGinty} = \big(\beta^2(p_C(6\,p_C - 6) + 1) + N^2(\beta - 2\,\beta\,p_C + 1)^2$$

$$- 2\,\beta\,N\,(\beta - p_C(\beta\,(5\,p_C - 5) + 2) + 1)\big)$$

$$/\Big(\beta^2 N\,\big((p_C(p_C(N-1) - 1) + 1)(N-1) + N^2$$

$$- \beta\,N\,p_C(2\,N - 2)\big)\Big). \qquad (31)$$

Given that we have checked our relatedness expression by simulation, this indicates that there is an error in Mc Ginty *et al.*'s (2013) relatedness expression. The simplest way to see that Mc Ginty *et al.*'s (2013) relatedness expression is erroneous is by substituting N=1 (one founder

cell per patch) into Equations 29 and 31. This should trivially lead to a relatedness of 1, because patches will comprise clones. This is the case for our relatedness expression (Equation 29), but not for Mc Ginty *et al.*'s (Equation 31).

However, despite the error in Mc Ginty *et al.*'s (2013) relatedness expression, we find that, in cases where the plasmid is very rare, very common, or very slowly transferred, Mc Ginty *et al.*'s (2013) relatedness expression converges on the correct relatedness expression (Equation 30). The results presented in the main text of Mc Ginty *et al.* (2013) were obtained by analysing the special cases where the plasmid is very rare or very slowly transferred. Therefore, fortunately, Mc Ginty *et al.*'s (2013) results are unaffected by their mathematical error in the derivation of the full plasmid relatedness expression.

Appendix C: Invasion criteria.

These derivations were first given in Dewar et al. (2021).

1) Invasion of a defector plasmid against non-cooperative hosts.

A defector plasmid (11) invades against a population of plasmid-free defector hosts (10) when the leading eigenvalue of the following matrix, evaluated at $x_{10}^* = 1, x_{11}^* = 0, x_{12}^* = 0,\ x_{20}^* = 0,\ x_{21}^* = 0, x_{22}^* = 0$, is greater than one:

$$\begin{pmatrix} \frac{\partial x_{10}''}{\partial x_{10}} & \frac{\partial x_{10}''}{\partial x_{11}} \\ \frac{\partial x_{11}''}{\partial x_{10}} & \frac{\partial x_{11}''}{\partial x_{11}} \end{pmatrix}.$$

This occurs when $\beta > \frac{C_C + s(1-C_C)}{\frac{N-1}{N}(1-s)(1-C_C)}$.

2) Invasion of a cooperator plasmid against cooperative hosts.

A cooperator plasmid (22) invades against a population of plasmid-free cooperative hosts (20) when the leading eigenvalue of the following matrix, evaluated at $x_{10}^* = 0, x_{11}^* = 0, x_{12}^* = 0$, $x_{20}^* = 1,\ x_{21}^* = 0, x_{22}^* = 0$, is greater than one:

$$\begin{pmatrix} \frac{\partial x_{20}''}{\partial x_{20}} & \frac{\partial x_{20}''}{\partial x_{22}} \\ \frac{\partial x_{22}''}{\partial x_{20}} & \frac{\partial x_{22}''}{\partial x_{22}} \end{pmatrix}.$$

This occurs when $\beta > \frac{C_C + s(1+B-C_C-C_G)}{\frac{N-1}{N}(1-s)(1+B-C_C-C_G)}$.

3) Invasion of a defector plasmid against cooperative hosts.

A defector plasmid (21) invades against a population of plasmid-free cooperative hosts (20) when the leading eigenvalue of the following matrix, evaluated at $x_{10}^* = 0, x_{11}^* = 0, x_{12}^* = 0,$ $x_{20}^* = 1,\ x_{21}^* = 0, x_{22}^* = 0$, is greater than one:

$$\begin{pmatrix} \frac{\partial x_{20}''}{\partial x_{20}} & \frac{\partial x_{20}''}{\partial x_{21}} \\ \frac{\partial x_{21}''}{\partial x_{20}} & \frac{\partial x_{21}''}{\partial x_{21}} \end{pmatrix}.$$

This occurs when $\beta > \frac{C_C + s(1+B-C_C-C_G)}{\frac{N-1}{N}(1-s)(1+B-C_C-C_G)}$. This is the same condition as the invasion of a cooperator plasmid against cooperative hosts.

4) <u>Invasion of a cooperator plasmid against non-cooperative hosts.</u>

A cooperator plasmid (12) invades against a population of plasmid-free cooperative hosts (10) when the leading eigenvalue of the following matrix, evaluated at $x_{10}^* = 1, x_{11}^* = 0, x_{12}^* = 0,$ $x_{20}^* = 0,\ x_{21}^* = 0, x_{22}^* = 0$, is greater than one:

$$\begin{pmatrix} \frac{\partial x_{10}''}{\partial x_{10}} & \frac{\partial x_{10}''}{\partial x_{12}} \\ \frac{\partial x_{12}''}{\partial x_{10}} & \frac{\partial x_{12}''}{\partial x_{12}} \end{pmatrix}.$$

This occurs when $\beta > \Big(2Bs - N - 2B + Ns + N^{\frac{1}{2}}\big(-(s-1)(4B + N - Ns + {C_C}^2N + {C_G}^2N - 2C_CN - 2C_GN + 2C_CC_GN + 2C_CNs + 2C_GNs - {C_C}^2Ns - {C_G}^2Ns - 2C_CC_GNs)\big)^{\frac{1}{2}} + C_CN + C_GN - C_CNs - C_GNs\Big) / \left(\frac{N-1}{N}(1-s)2B\right)$.

5) <u>Region of parameter space where a cooperator plasmid has a (transient) selective advantage over a cheat plasmid (i.e., a selective advantage whilst plasmids are rare).</u>

When plasmids are rare, relatedness at the plasmid locus is given by $R_{RarePlasmid} = \frac{1}{N}\frac{(N+(N-1)\beta)^2}{N^2+(N-1)\beta\text{^}2}$. This expression was first derived by Mc Ginty *et al.* (2013). Whilst plasmids are rare, the cooperator plasmid has a selective advantage over cheat plasmids whenever $R_{RarePlasmid}B > C_G$ is satisfied. This condition can be written explicitly in terms of model parameters as: $\frac{1}{N}\frac{(N+(N-1)\beta)^2}{N^2+(N-1)\beta^2}B > C_G$.

Appendix D: Plasmids versus greenbeards.

Greenbeard genes are genes that can recognise copies of themselves in other individuals, and then help and receive help from those individuals (Gardner & West, 2010; Madgwick *et al.*, 2019). Greenbeard genes provide a possible way for cooperation to be favoured, in addition to standard genealogical relatedness (kin selection) and horizontal gene transfer. By only interacting with other individuals who also have the greenbeard gene, relatedness for greenbeard-encoded helping may be maximal (=1), meaning Hamilton's Rule has a good chance of being satisfied, leading to the greenbeard being favoured.

Greenbeards may help obligately or facultatively (Gardner & West, 2010). *Facultative helping greenbeards* only provide help when they come across another individual with the same greenbeard gene. *Obligate helping greenbeards* always pay a cost of helping, even if there are no greenbeard-matching individuals around to make use of that help. *Obligate helping greenbeards*, unlike *facultative helping greenbeards*, are disfavoured below a certain frequency (positive frequency dependence) (Gardner & West, 2010). *Obligate helping greenbeards* are disfavoured below a certain frequency because there are few greenbeard-matching individuals around to receive the help, meaning the helping effort is wasted. On this basis, some have argued that *obligate helping greenbeards* should be rare in nature, because they cannot reach a high enough frequency to become favoured.

However, the "positive frequency dependence" argument for the rarity of obligate helping greenbeards is undermined by the possibility of population structure (Rousset, 2004; Gardner & West, 2010). Specifically, population structure can allow obligate helping greenbeards to be locally common, even though they are globally rare, removing the positive frequency dependent selection, and allowing them to be selected even at low global frequencies.

A reviewer wondered whether our argument, for why plasmid-mediated cooperation should be generally low, is analogous to the “positive frequency dependence” argument for why obligate helping greenbeards are rare. Furthermore, they wondered whether, like the “positive frequency dependence” argument, our argument about plasmid-mediated cooperation may be undermined by the possibility of population structure.

Fortunately, the possibility of population structure does not undermine our argument, for two reasons. Firstly, unlike obligate helping greenbeards, there is no positive frequency dependent selection on cooperative plasmids – the cooperative plasmids do not need to be above a certain frequency before they are favoured. In fact, the opposite is true – cooperative plasmids are most favoured at low frequencies, where plasmid relatedness is highest. It is *negative frequency dependence* that stops cooperative plasmids from reaching high frequencies and having an appreciable impact on bacterial population. Secondly, we have already examined population structure (population structure is given by the inverse of the number of founders, N, per patch), and found it to not qualitatively affect results in general.

Appendix E: Equilibria and non-equilibria.

We have often discussed evolutionary equilibria, even though in nature many plasmids will be in continuous flux, and never settle down to an evolutionary equilibrium. An additional reason for focusing on equilibria is that it is more natural to make points about the long-term steady state of a system (which is unique), rather than short-term states (which are unpredictable, being contingent on initial conditions). This is a pragmatic argument for referring to equilibria (which are knowable) rather than nonequilibrium states (which are generally not knowable, unless initial conditions are fixed).

However, we stress that we did not *exclusively* focus on equilibria. Indeed, an important part of our argument is about evolutionary trends that occur away from equilibrium states. For instance, we showed that cooperative plasmids often gain a transient advantage, allowing them to invade, before losing this advantage as plasmids reach high population frequency, causing them to ultimately be lost from the population. This is a detailed characterisation of non-equilibrium dynamics, and gives insight into why the final equilibrium (loss of the cooperative plasmid) comes about.

Supplementary figures

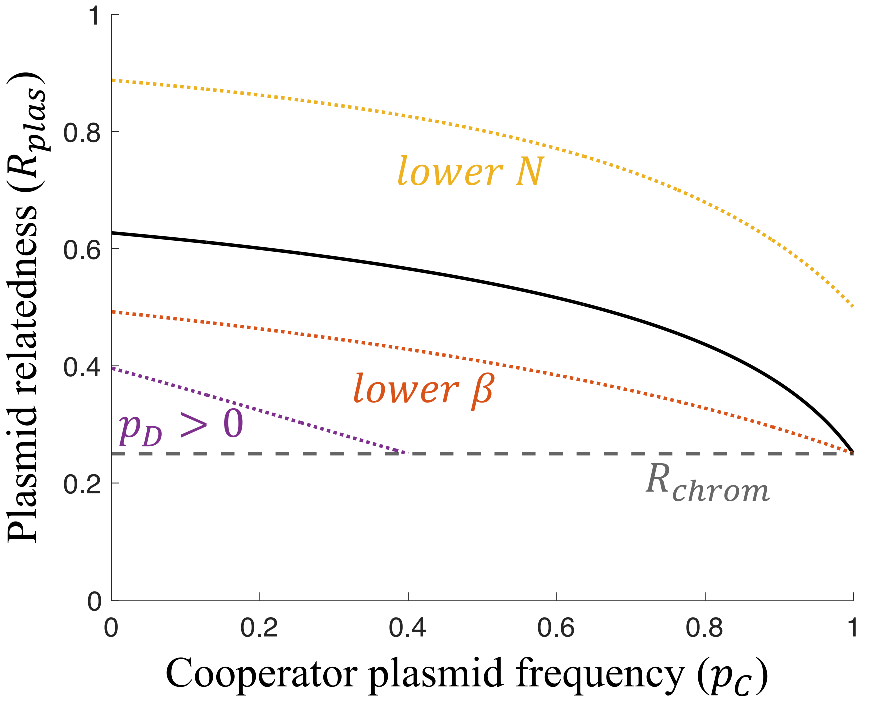

Supplementary Figure 1. Plasmid relatedness. We plot plasmid relatedness (R_{plas}; Equation 29) as a function of the population frequency of the cooperator plasmid (p_C), for different: numbers of founder cells per group (N); plasmid transfer probabilities (β); defector plasmid frequencies (p_D). The chromosome relatedness (R_{chrom}) is equal to $1/N$ and plotted for N=4 (dashed black line). The solid black line represents the case without defector plasmids (p_D=0), β=0.95, and N=4. The coloured dashed lines represent scenarios where there is a lower number of founder cells (N=2; yellow line), a lower plasmid transfer probability (β=0.6; red line), or some defector plasmids (p_D=0.6; purple line). When plasmids are at fixation (p_C+p_D=1), plasmid relatedness (R_{plas}) equals chromosome relatedness (R_{chrom}).

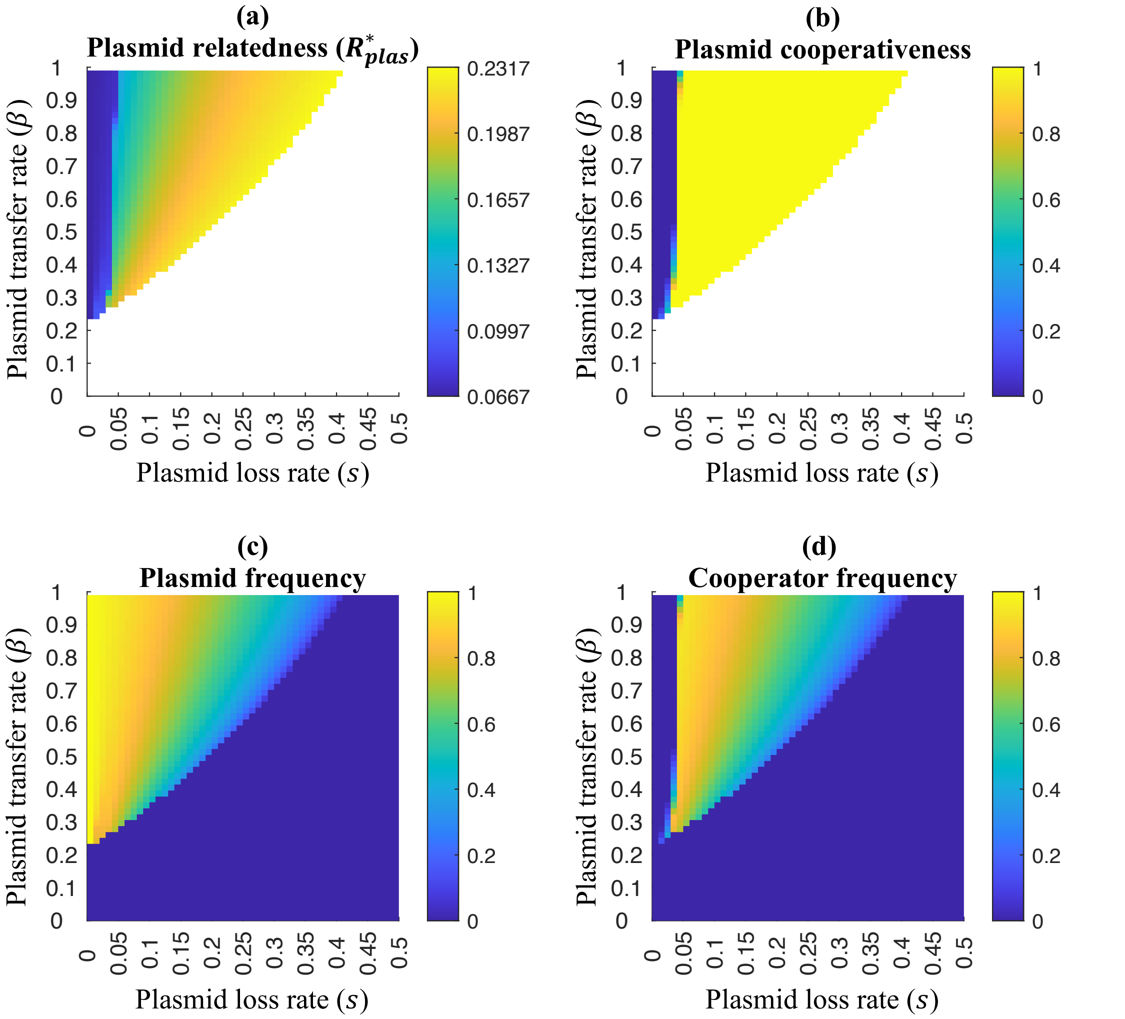

Supplementary Figure 2. Reducing the number of founders (N) facilitates plasmid-mediated cooperation. Example results for a region of parameter space where cooperation is not favoured at the chromosome (individual) level ($R_{chrom}B<C_G$), meaning cooperation only evolves if it is encoded by a plasmid. We assumed the same parameters as Figure 2, except for a reduced number of founders: N=15, C_C=0.2, C_G=0.1, B=1.435. For this number of founders (N=15), cooperation is only slightly disfavoured favoured at the individual (chromosome) level ($R_{chrom}B$=0.096, C_G=0.1), meaning a relatively small increase in plasmid relatedness (R_{plas}) relative to chromosomal relatedness (R_{chrom}) will be sufficient for cooperation to evolve. We record the equilibrium: (**A**) plasmid relatedness (Equation 29); (**B**) plasmid cooperativeness (ratio of cooperator to defector plasmids; we find that this is 1 for $R^*_{plas}B > C_G$ and 0 for $R^*_{plas}B < C_G$); (**C**) plasmid frequency (including both cooperator and defector plasmids); (**D**) cooperator frequency (given by the product of plasmid cooperativeness and frequency). The white areas in A and B represent areas of parameter space that are undefined because the plasmid is absent. The lowest recorded plasmid relatedness in A is equal to chromosomal relatedness (0.0667=1/N).

Supplementary information references

Birch, J. (2014) Gene mobility and the concept of relatedness. *Biol Philos*, **29**, 445–476.

Birch, J. (2017) *The Philosophy of Social Evolution*. Oxford University Press.

Frank, S.A. (1998) *Foundations of Social Evolution*. Princeton University Press.

Gardner, A. & West, S.A. (2010) Greenbeards. *Evolution*, **64**, 25–38.

Gardner, A., West, S.A. & Wild, G. (2011) The genetical theory of kin selection. *Journal of Evolutionary Biology*, **24**, 1020–1043.

Grafen, A. (1985) A geometric view of relatedness. *Oxford surveys in evolutionary biology*, **262**, 391–397.

Madgwick, P.G., Belcher, L.J. & Wolf, J.B. (2019) Greenbeard Genes: Theory and Reality. *Trends in Ecology and Evolution*, **34**, 1092–1103.

Mc Ginty, S.É., Lehmann, L., Brown, S.P. & Rankin, D.J. (2013) The interplay between relatedness and horizontal gene transfer drives the evolution of plasmid-carried public goods. *Proceedings of the Royal Society B: Biological Sciences*, **280**, 20130400.

Pepper, J.W. (2000) Relatedness in Trait Group Models of Social Evolution. *Journal of Theoretical Biology*, **206**, 355–368.

Rankin, D.J., Rocha, E.P.C. & Brown, S.P. (2011) What traits are carried on mobile genetic elements, and why? *Heredity*, **106**, 1–10.

Rousset, F. (2004) *Genetic Structure and Selection in Subdivided Populations (MPB-40)*. Princeton University Press.

Rousset, F. (2015) Regression, least squares, and the general version of inclusive fitness. *Evolution*, **69**, 2963–2970.

Scott, T.W., West, S.A., Dewar, A.E. & Wild, G. (2023) Is cooperation favoured by horizontal gene transfer? *GitHub, https://doi.org/10.5281/zenodo.7585371.*
